# Glucose but Not Fructose Alters the Intestinal Paracellular Permeability in Association With Gut Inflammation and Dysbiosis in Mice

**DOI:** 10.3389/fimmu.2021.742584

**Published:** 2021-12-27

**Authors:** Xufei Zhang, Magali Monnoye, Mahendra Mariadassou, Fabienne Beguet-Crespel, Nicolas Lapaque, Christine Heberden, Veronique Douard

**Affiliations:** ^1^ Université Paris-Saclay, INRAE, AgroParisTech, MICALIS Institute, Jouy-en-Josas, France; ^2^ Université Paris Saclay, INRAE, MaIAGE, Jouy-en-Josas, France

**Keywords:** glucose, fructose, Caco-2, *Desulfovibrio*, paracellular permeability, pro-inflammatory cytokines

## Abstract

A causal correlation between the metabolic disorders associated with sugar intake and disruption of the gastrointestinal (GI) homeostasis has been suggested, but the underlying mechanisms remain unclear. To unravel these mechanisms, we investigated the effect of physiological amounts of fructose and glucose on barrier functions and inflammatory status in various regions of the GI tract and on the cecal microbiota composition. C57BL/6 mice were fed chow diet and given 15% glucose or 15% fructose in drinking water for 9 weeks. We monitored caloric intake, body weight, glucose intolerance, and adiposity. The intestinal paracellular permeability, cytokine, and tight junction protein expression were assessed in the jejunum, cecum, and colon. In the cecum, the microbiota composition was determined. Glucose-fed mice developed a marked increase in total adiposity, glucose intolerance, and paracellular permeability in the jejunum and cecum while fructose absorption did not affect any of these parameters. Fructose-fed mice displayed increased circulation levels of IL6. In the cecum, both glucose and fructose intake were associated with an increase in *Il13*, *Ifnγ*, and *Tnfα* mRNA and MLCK protein levels. To clarify the relationships between monosaccharides and barrier function, we measured the permeability of Caco-2 cell monolayers in response to IFNγ+TNFα in the presence of glucose or fructose. *In vitro*, IFNγ+TNFα-induced intestinal permeability increase was less pronounced in response to fructose than glucose. Mice treated with glucose showed an enrichment of *Lachnospiracae* and *Desulfovibrionaceae* while the fructose increased relative abundance of *Lactobacillaceae*. Correlations between pro-inflammatory cytokine gene expression and bacterial abundance highlighted the potential role of members of *Desulfovibrio* and *Lachnospiraceae NK4A136 group* genera in the inflammation observed in response to glucose intake. The increase in intestinal inflammation and circulating levels of IL6 in response to fructose was observed in the absence of intestinal permeability modification, suggesting that the intestinal permeability alteration does not precede the onset of metabolic outcome (low-grade inflammation, hyperglycemia) associated with chronic fructose consumption. The data also highlight the deleterious effects of glucose on gut barrier function along the GI tract and suggest that *Desulfovibrionaceae* and *Lachnospiraceae* play a key role in the onset of GI inflammation in response to glucose.

## Introduction

Increase in consumption of sugar (mainly sucrose and high fructose corn syrup-containing soft drinks) has been tightly linked to the surge in metabolic diseases such as obesity, non-alcoholic fatty liver disease, hypertriglyceridemia, type 2 diabetes, and metabolic syndrome (1–6). Sucrose and high-fructose corn syrup are both composed of near-equal amounts of glucose and fructose. There is rising evidence that in these sweeteners, fructose and, in a lesser extent, glucose favor the development of metabolic diseases associated with sugar intake (7–10). Obesity-associated chronic low-grade inflammation characterized by high levels of plasmatic inflammatory markers [C-reactive protein, interleukin 6 (IL6) or tumor necrosis factor α (TNFα)] largely contributes to the development of the obesity-related chronic metabolic diseases (11–13). The origins of the low-grade inflammation observed in metabolic disorders are still not entirely understood. However, recent work showed that the gut contributes to its establishment (12, 14). Gut barrier defects (increased permeability, mucosal inflammation and dysbiosis) favor the translocation of microbial products, which promulgate inflammation to the peripheral tissues (15, 16). To maintain the intestinal barrier function, the preservation of the paracellular permeability is essential. The tight junctions (TJ) primarily regulate the barrier formed between epithelial cells apically. Occludin, claudins, junctional adhesion molecules (JAMs), and zonula occludens (ZO) are the main TJ proteins that form the TJ complex. The intestinal paracellular permeability depends on TJ protein expression levels, their phosphorylation status, and their subcellular organization (17–19). While the events involved in the loss of the barrier function are not fully understood, cytokines such as IL1β, IL13, interferon gamma (IFNγ), and TNFα have been shown to remodel the TJ architecture and thus increase paracellular permeability (20–22). Other factors, such as the enteric nervous system (ENS), also participate in the regulation of the intestinal permeability. The ENS is constituted of enteric neurons, which are surrounded by large populations of enteric glial cells (EGCs). Several studies suggested that glial fibrillary acidic protein (GFAP)-positive EGCs participate in the regulation of the inflammatory response and in the regulation of the integrity of the gut epithelium (23).

Although less well explored than in the context of high fat diet, the role of intestinal permeability is also suspected to be essential for the development of metabolic disorders resulting from high sugar intake. Luminal glucose has long been known to regulate intestinal paracellular permeability *in vitro* in Caco-2 cells (24), and *in vivo* in the jejunum of rats (25, 26) and humans (27). Recently, in the context of hyperglycemia, plasma glucose has been demonstrated to alter the transcription of TJ proteins and intestinal permeability *via* its retro-transport into enterocytes by the basolateral glucose transporter GLUT2 (28). The effect of dietary fructose on intestinal permeability is less clear and the association of its high intake with systemic inflammation has been inconsistent (29, 30). Fructose consumption has been reported to decrease occludin protein levels in the mouse small intestine (31, 32) and has been associated with endotoxemia, suggesting an altered intestinal barrier function (32, 33). However, the direct effect of fructose-induced alteration of intestinal barrier function on the onset of metabolic outcome in response to chronic fructose intake remains unexplored. Indeed, fructose impact on intestinal permeability has scarcely been studied and often in association with high levels of dietary lipids (34) or non-physiological amounts of fructose (29). Since fructose is also associated with increase in glycemia (29, 35), which is associated with increase in intestinal permeabilibity in a GLUT2-dependent manner (28), the direct role of luminal fructose on the gut barrier function must be questioned.

In this study, using dietary glucose as a positive control, we aim to clarify the impact of fructose on the intestinal permeability prior to the onset of hyperglycemia. Fructose absorption has been reported to take place in the small intestine (36). However, a recent study showed that high fructose intake can overwhelm the sugar absorptive capacity of the small intestine and non-absorbed fructose can reach the lower intestine (37). Therefore, we assessed the effects of chronic intake of physiological amounts of fructose on the paracellular permeability of the small intestine, the cecum, and the colon. In order to clarify the mechanism involved, we evaluated changes in gut microbiota composition, expression of TJ proteins, and inflammatory and ENS markers. Our results demonstrate that fructose does not increase the intestinal permeability in the small intestine where its luminal concentration is the highest. Moreover, while the inflammation of the cecum was similar in fructose- and glucose-fed mice, the intestinal paracellular permeability increased only in response to chronic intake of glucose, but not of fructose. Finally, we showed that in an *in vitro* Caco-2 cell model, glucose but not fructose enhances inflammation-induced permeability increase.

## Materials and Methods

### Animals and Experimental Design

This study was conducted in accordance with French guidelines on animal experimentation and validated by the Ethics Committee in Animal Experiment of INRA Jouy-en-Josas (Comethea, registration number: APAFIS#1620-2015102618572930v2). Male C57BL/6NRj mice (7 weeks old, Janvier Labs, St Bertevin, France) were individually housed and maintained in 12-h light/12-h dark cycle. Mice received, before and during the experimental procedure, a chow diet meeting their complete need (Ssniff, Soest, Germany). After 2 weeks of adaptation, the mice received as drinking solution either water (Control), 15% glucose (Glucose), or 15% fructose (Fructose) for 9 weeks. The duration and sugar concentration were based on previous studies showing modest metabolic outcomes in response to 15% fructose (38–40). Food and water were provided *ad libitum*. Body weight, drink, and food intakes were measured three times per week. Caloric intake measurement was calculated based on the daily average intake (in grams and milliliters) of chow (3.27 kcal/g of chow) and drinking solution (0.6 kcal/ml of glucose or fructose solution), respectively. After 9 weeks, non-starved mice were euthanized. Cardiac blood was collected under anesthesia into EDTA tubes, centrifuged (4°C, 2,000*g*, 15 min), and stored at −80°C for cytokine analysis. Non-flushed 2-cm sections of jejunum, cecum, and colon were sampled and stored in Krebs buffer for instant intestinal permeability measurement in Ussing chambers. Flushed sections of jejunum, cecum, and colon were sampled, flash frozen in liquid nitrogen, and stored at −80°C for protein and mRNA analysis. The cecal content was sampled and stored at −80°C for bacterial DNA analysis. Liver was sampled and weighed.

### Oral Glucose Tolerance Test

Oral glucose tolerance test (OGTT) was performed after 8 weeks of exposure to 15% glucose or 15% fructose beverage. Glucose was orally administered (2 g/kg body weight) to the mice after 12 h fasting, during which food as well as fructose- or glucose-drinking solutions were removed and replaced with water.

### Ussing Chamber Permeability Measurement

Shortly after sampling, intestinal sections of jejunum, cecum, and colon were opened along the mesenteric border and mounted in Ussing chambers (Physiologic Instruments, San Diego, USA) using an appropriate slider area size (0.2 cm^2^ for jejunum and cecum, 0.1 cm^2^ for colon). The mucosal side of the tissue was exposed to oxygenated Krebs-mannitol (10 mM) buffer, while the serosal side was exposed to oxygenated Krebs-glucose (10 mM) buffer. All buffers were maintained at 37°C. Fluorescein isothiocyanate-sulfuric acid (FITC-SA; Thermo Fisher Scientific, France) was used as a marker of TJ paracellular permeability. After adding FITC-SA (40 µg/ml) to the mucosal chamber, 100 µl was collected in duplicate from the serosal chamber every 15 min for 2 h. The concentration of FITC-SA was measured *via* fluorescence at excitation 485 nm and emission 538 nm. Paracellular permeability was measured by the flux of FITC-SA expressed as ng/cm^2^/h.

### RNA Extraction and Quantitative Real-Time PCR

The total mRNA from jejunum, cecum, and colon sections were extracted using MirVana miRNA isolation kit (Thermo Fisher Scientific, France). Two micrograms of mRNA was reverse transcribed using the High Capacity Complementary DNA Reverse Transcription Kit (Thermo Fisher Scientific, France) as described previously (41). Quantitative real-time PCR (qRT-PCR) was performed using SYBR-Green^®^ PCR 2X Master Mix (Applied-Biosystems, France) to quantify the gene expression of TJ proteins and inflammatory markers. β-actin was used as a housekeeping gene to normalize the mRNA abundance of each target gene (primers are listed in **Table S1**). Expression values of target genes were calculated based on the comparative threshold cycle (Ct) method to generate ΔCt values. The relative abundance of each mRNA in each sample was then normalized according to the equation: Relative Quantity RQ = 2^−ΔΔCt.^

### Protein Extraction and Western Blot

Total protein from cecum sections was extracted using RIPA buffer (Sigma-Aldrich, France) with 4% and 10% protease inhibitor (Roche, Sigma-Aldrich, France) and phosphatase (Roche, Sigma-Aldrich, France), respectively, and quantified using the RC DC Protein Assay kit (Biorad, France). Western blot analyses were performed using 50 μg of intestine and membranes were probed with polyclonal antibodies against myosin light-chain kinase (MLCK) (Sigma-Aldrich, France) diluted 1:2,000 and then stripped and reprobed with mouse anti-GAPDH (glyceraldehyde 3-phosphate dehydrogenase) antibody (Sc-365062 Santa Cruz, USA). The blots were revealed using enhanced chemiluminescent horseradish peroxidase substrate (ECL-HRP Thermo Fisher Scientific, France) and visualized using ChemiDoc MP imaging system (Bio-Rad, France). All bands were digitally captured and densitometry analysis was performed using image Lab 5.0 software (Bio-Rad, France).

### Immunoprecipitation of GFAP

Three hundred micrograms of cecum protein extract was incubated for 2 h at RT and then overnight at 4°C with rabbit monoclonal anti-GFAP antibody (1:40, Ab68428, Abcam, France). The immune complexes were captured with 50% Protein A Agarose beads (Cell Signaling, Saint Quentin Yvelines, France) on a rotator for 2 h at RT. After centrifugation (4°C, 20,000*g*, 5 min), the precipitates were washed three times with RIPA buffer. The final pellets were suspended in 30 µl of 2× loading buffer and incubated at 95°C for 15 min. The entire suspension was loaded onto a SDS-PAGE gel, and the Western blot for GFAP (1:10,000) was performed as described above and using peroxidase-labeled secondary anti-rabbit antibody (1:400, Abcam, France). Blot image analysis was performed as described above.

### Plasma Analysis

IL6, TNFα, and IFNγ levels were determined by ELISA (Peprotech, France) using 50 µl of plasma diluted 1:2.

### Caco-2 Cell Culture and TEER Measures *In Vitro*

Caco-2BBe cells (kindly donated by JR. Turner, Harvard Medical School) and cultured in high-glucose (25 mM) Dulbecco’s modified Eagle’s medium (DMEM, Gibco, Thermo Fisher Scientific, France) with 10% fetal bovine serum (FBS, Sigma-Aldrich, France) and 10 ng/ml human transferrin. Caco-2BBe cells were seeded at 2 × 10^5^ cells/well and differentiated as monolayers on collagen-coated polycarbonate membrane transwells with 0.3-μm pores (Thermo Fisher Scientific, France) for 15–18 days after confluence. DMEM medium was renewed every 3 days. Transepithelial resistance (TEER) was measured every 3 days with Voltohmmeter instrument (Millicell-ERS, Merck Millipore, France). In experiment 1, after 15–18 days (when TEER was about 250 Ω.cm^2^ and stable), the culture medium was changed to 25 mM glucose or 25 mM fructose medium with 10% FBS and 10 ng/ml transferrin in both basal and apical chambers 1-day prior to cytokine treatment (D0). In experiment 2, to determine whether glucose and fructose were necessary for IFN-γ/TNF-α-induced changes in TEER, at D0, the 25 mM glucose medium was changed to 1 mM glucose or 1 mM fructose medium. At D1, recombinant human IFN-γ (Peprotech, France) was added into glucose or fructose medium (10 ng/ml concentration) in the basal chamber for 24 h. At D2, new medium containing 50 ng/ml TNF-α (Peprotech, France) was placed in the basal chamber. Both IFN-γ and TNF-α treatments were done without manipulating the apical chamber. The TEER was measured before adding TNF-α and every hour afterward for 6 h. Results were expressed as percent of TEER of control monolayers (cells exposed to glucose or fructose for 48h without IFN-γ and TNF-α) of the same experiment.

### Microbiota DNA Extraction and 16S DNA Sequencing

Total DNA was extracted from cecal content samples using GNOME DNA isolation kit (MP Biomedicals, Strasbourg, France). The V3–V4 hypervariable region of the bacterial 16S rDNA was amplified by PCR (forward primer: 5’-CTTTCCCTACACGACGCTCTTCCGATCTACGGRAGGCAGCAG-3’, reverse primer: 5’-GGAGTTCAGACGTGTGCTCTTCCGATCTTACCAGGGTATCTAATCCT-3’; 94°C for 1 min and then 30 cycles at 94°C for 1 min, 65°C for 1 min, and 72°C for 1 min before a final step at 72°C for 10 min) (42). After quality checking by electrophoretic 2% agarose gel migration, obtained amplicons were sequenced using Illumina MiSeq technology (GenoToul platform, Toulouse, France). Paired-end reads obtained from MiSeq sequencing were analyzed using the Galaxy-supported pipeline named FROGS [Find, Rapidly, OTUs (operational taxonomic units) with Galaxy Solution] (43). For the preprocessing, reads with length ≥ 380 bp were kept. The clustering and chimera removal tools followed the guidelines of FROGS (43). Assignment was performed using SILVA 16 S. OTUs with abundances lower than 0.005% were removed from the analysis (44). Real-time PCR analyses were performed to quantify *Desulfovibrio* genus abundance. Quantitative PCR reactions were conducted in a final volume of 25 μl using SYBR-Green^®^ PCR 2X Master Mix (Applied-Biosystems, France) and with 0.2 μM final concentration of each primer (DSV691-F CCGTAGATATCTGGAGGAACATCAG and DSV826-R: ACATCTAGCATCCATCGTTTACAGC) and 5 μl of DNA samples (45).

### Bacteria *In Vitro* Culture

*Bacteroides vulgatus* (ATCC 8482) and *Lactobacillus johnsonii* (strain CIP 103620) were grown in DP2 growth medium enriched with 5 g/L of glucose or 5 g/L of fructose (46). *Desulfovibrio vulgaris* subsp. *vulgaris* (DSM 644) was grown in appropriate Medium 63 containing either glucose (5 g/L) or fructose (5 g/L). All bacteria were cultured in anaerobic condition at 37°C using the Hungate culture method. For each bacterial species, 24 h growth media (containing no fructose) were inoculated with a 1:9 dilution to a duplicate set of young culture containing glucose or fructose. The growth of *B. vulgatus* and *L. johnsonii* was measured every 30 min or 1 h by absorbance (A660) for 8–10 h. For *D. vulgaris* after 4 h and 9 h, the DNA was extracted using GNOME DNA isolation kit (MP Biomedicals, Strasbourg, France) and the growth was measured by qPCR using the primers DSV691-F CCGTAGATATCTGGAGGAACATCAG and DSV826-R: ACATCTAGCATCCATCGTTTACAGC. The total number of bacteria was inferred from averaged standard curves as previously described (47).

### Statistical and Microbiota Analysis

Statistical analysis was performed using GraphPad Prism software (v7; San Diego, CA). All data were analyzed using Kruskal–Wallis test followed by a Dunn’s multiple comparison test. 16S rDNA sequencing data were analyzed using the Phyloseq package in R and custom scripts as described previously (48). Briefly, 16S sequencing data were analyzed using the Phyloseq (49), ggplot2 (50), DESeq2 (51), and custom scripts. Samples were rarefied to even sampling depths before α-diversities (observed richness and Inverse Simpson) and β-diversity (Bray–Curtis) analysis. Principal coordinates analysis (PCoA) was performed on Bray–Curtis dissimilarities. α-diversity data were analyzed using Kruskal–Wallis test. Permutational multivariate analysis of variance (PERMANOVA) test was performed on the Bray–Curtis matrices using 999 random permutations and at a significance level of 0.01. Phylum and family abundances data were compared using Kruskal–Wallis test. Raw, unrarefied OTU (also named clusters) counts were used to produce relative abundance graphs and to find taxa with significantly different abundances between two groups. DESeq2 was used to estimate abundance log-fold changes (logfc) between glucose/control, fructose/control, or glucose/fructose groups. Clusters were selected based on effect size (fold change (FC) > 1.5 or FC < 1/1.5) and adjusted *p*-value (<0.05). Pro-inflammatory cytokine gene expression levels (TNFα, IL13, Il22, and IFNγ) and normalized bacterial abundance (52) were integrated and analyzed using Data Integration Analysis for Biomarker discovery using a Latent cOmponents (DIABLO) method implemented in R mixOmics package (53). Correlation analysis between cytokines gene expression and bacterial species abondance was performed using the multiblock analysis DIABLO (block.splsda function) (54). Horizontal sparse partial least squares-discriminant analysis (sPLS-DA) was used to integrate the relative abundances of OTU (clusters) with the cytokine gene expression levels. To perform the analysis, 5% of the OTU (which were the most involved in the group discrimination) were selected. The level of correlation between the OTU and the cytokine gene expression level was determined and the resulting correlation network showing only positive (>0.83) and negative (<−0.83) correlations between the selected variables was built in DIABLO.

## Results

### Glucose and Fructose Modified Differently Physiological Parameters

**Figure 1:**
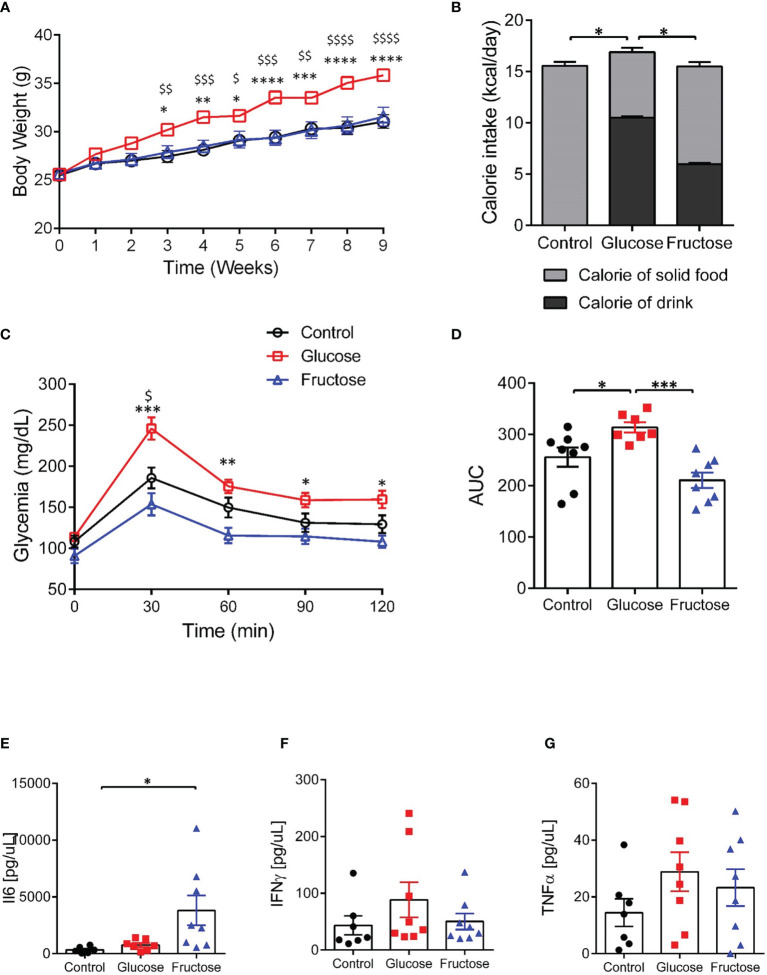
Effects of glucose and fructose on body and plasma parameters. **(A)** Body weight changes during 9 weeks of exposure to glucose or fructose. **(B)** Average daily caloric intake originating from solid diet or drink solutions. **(C)** Blood glucose levels during oral glucose tolerance tests. **(D)** Area under the curve (AUC) of blood glucose levels during OGTT. Plasma levels of **(E)** IL6, **(F)** INFγ, and **(G)** TNFα. All values are means ± SEM; *n* = 6–8/group. For figures A and C: At each time point, mean data were compared by Kruskal–Wallis test followed by Dunn multiple comparison test. For figures A and C, * indicates a significant difference between glucose and fructose groups (*p < 0.05; **p < 0.01; ***p < 0.001; ****p < 0.0001) while ^$^ indicates a significant difference between glucose and control groups (^$^ *p* < 0.05; ^$$^ *p* < 0.01; ^$$$^ *p* < 0.001; ^$$$$$^ *p* < 0.0001). For the other figures, means were compared by Kruskal–Wallis test followed by Dunn multiple comparison test and * indicates a significant difference among the groups (*p < 0.05; **p < 0.01).

Mice-fed glucose solution gained 15% more weight than the fructose- and water-drinking mice after 9 weeks of experiment (**Figure 1A**). This likely resulted from a significantly higher total daily calorie intake by the glucose-drinking mice when compared to mice receiving fructose or water solutions (**Figure 1B**). Eight weeks of chronic intake of glucose impaired glucose tolerance both in the individual points of the curve (**Figure 1C**) and in the AUC (**Figure 1D**) when compared to control and fructose groups. Nine weeks of chronic intake of fructose was associated with a significant increase in plasmatic levels of IL6 when compared to the control group (**Figure 1E**) while the plasmatic levels of TNFα and IFNγ remained unchanged among the three groups (**Figures 1F, G**). After 9 weeks, glucose-drinking mice also displayed a significantly higher visceral adiposity (2.94% ± 0.19) than the fructose-drinking (1.66% ± 0.22) or control (1.52% ± 0.14) groups. There was no difference in liver weight among the three groups (data not shown). Despite a major effect of glucose intake on body weight gain and glucose tolerance, only fructose initiated an increase in circulating levels of pro-inflammatory cytokines.

### Glucose But Not Fructose Altered Intestinal Permeability

**Figure 2:**
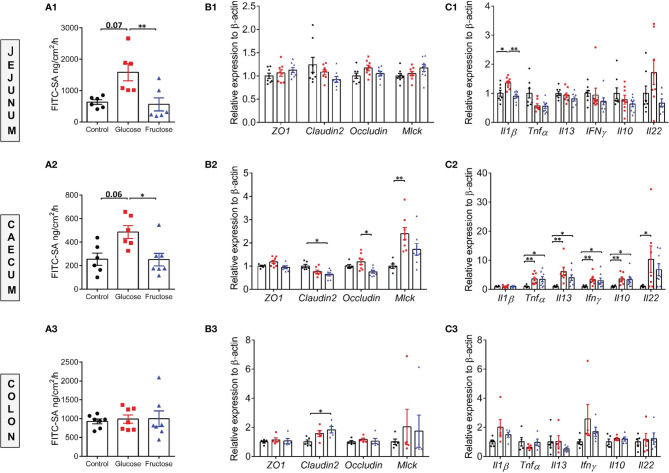
Paracellular permeability, intestinal permeability, and inflammatory marker expression levels in various regions of the gut. *Ex vivo* paracellular permeability to FITC-sulfonic acid measured in the jejunum **(A1)**, cecum **(A2)**, and colon **(A3)**. The mRNA expression levels of gene encoding for tight junction proteins in the jejunum **(B1)**, cecum **(B2)**, and colon **(B3)**. The mRNA expression levels of inflammatory cytokine markers measured in the jejunum **(C1)**, cecum **(C2)**, and colon **(C3)**. The mRNA level data were normalized to the control group. All values are means ± SEM; *n* = 5–8/group. Means were compared by Kruskal–Wallis test followed by Dunn multiple comparison test. * indicates significant differences among groups (*p < 0.05 and **p < 0.01). FITC-SA: Fluorescein isothiocyanate-sulfuric acid; *Il1*β: Interleukin 1 beta; *Il10*: Interleukin 10; *Il13*: Interleukin 13; *Il22*: Interleukin 22; *Ifnγ*: Interferon gamma; *Mlck*: Myosin light-chain kinase; *ZO-1*: Zonula occludens-1; *Tnfα*: Tumor necrosis factor alpha.

**Figure 3:**
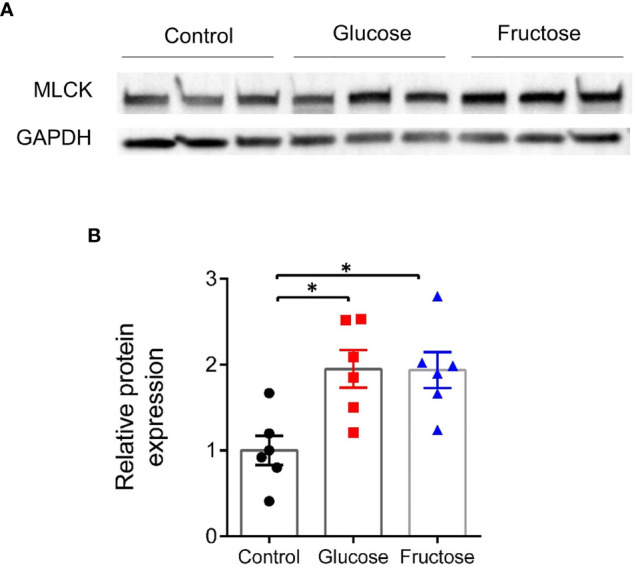
Protein expression level of MLCK in the cecum. **(A)** Representative images of Western blots for MLCK. **(B)** Relative band intensity of MLCK normalized to GAPDH and expressed relative to the control group. All values are means ± SEM; *n* = 6/group. Means were compared by Kruskal–Wallis test followed by Dunn multiple comparison test. * indicates significant differences among groups (* *p* < 0.05). MLCK, Myosin light-chain kinase; GAPDH, Glyceraldehyde 3-phosphate dehydrogenase.

Earlier studies suggested that barrier defects contribute to the establishment of dietary-associated chronic low-grade inflammation (55, 56). To determine whether fructose-induced systemic inflammation was associated with alteration of intestinal permeability, we evaluated this parameter in the small (jejunum) and large intestine (cecum and proximal colon) *ex vivo* using the Ussing chamber system. Nine weeks of glucose consumption significantly increased paracellular permeability to FITC-SA in both jejunum and cecum when compared to the fructose groups (**Figures 2A1, A2**). Glucose intake also lead to a trend toward increased permeability in jejunum and cecum when compared to the control group. In the colon, neither glucose nor fructose changed the paracellular permeability (**Figure 2A3**). In the jejunum, glucose-induced changes in permeability were not associated with changes in *Mlck* and TJ protein expression levels (**Figure 2B1**). Of the pro-inflammatory cytokines tested, only *Il1β* expression was significantly upregulated in the jejunum of the mice consuming glucose when compared to the control and fructose groups (**Figure 2C1**). In the cecum, glucose-induced increase in permeability was associated with a significant increase in *Mlck* expression when compared to the control group (**Figure 2B2**). In contrast, fructose-drinking mice displayed a significant decrease in expression of *claudin2* and *occludin* relative to the control and glucose groups respectively. In cecum, both glucose and fructose significantly increased the expression of *Tnfα, Il13*, *Ifnγ*, and *Il10* when compared to the control group (**Figure 2C2**). In addition, glucose intake was also associated with a significant increase in *Il22* expression in the cecum when compared to the mice of the control group. Only *Il1β* expression remained unchanged among the three groups. In the colon, fructose moderately increased *claudin2* expression while the expression of the other TJ proteins remained unchanged in response to glucose or fructose (**Figure 2B3**). Likewise, none of the pro-inflammatory cytokines tested displayed any changes in expression among the three groups (**Figure 2C3**). *Il6* expression levels remain unchanged among the three groups in the jejunum, cecum, and ileum (data not shown). Western blot showed that in the cecum, MLCK protein level increased significantly in response to glucose and fructose (**Figure 3**). Thus, in the cecum, both glucose and fructose caused similar upregulation of pro-inflammatory cytokine expression and MLCK protein levels, but only glucose was associated with increased permeability.

In addition to the above gut homeostasis effects, glucose and fructose intake significantly upregulated in jejunum the expression of the apical sodium/glucose cotransporter, *Sglt1*, and of the basolateral glucose transporter, *Glut2*, but only fructose drastically upregulated the expression of its apical transporter, *Glut5* (**Figure S1A**). In the cecum, glucose intake was associated with an increase in expression levels of both glucose transporters, *Sglt1* and *Glut2*, while the fructose intake did not change *Glut5, Sglt1*, or *Glut2* mRNA levels (**Figure S1B**). These data suggest that glucose and fructose transport levels are likely different between the jejunum and cecum.

### Fructose, But Not Glucose, Affected the EGC of the ENS

**Figure 4:**
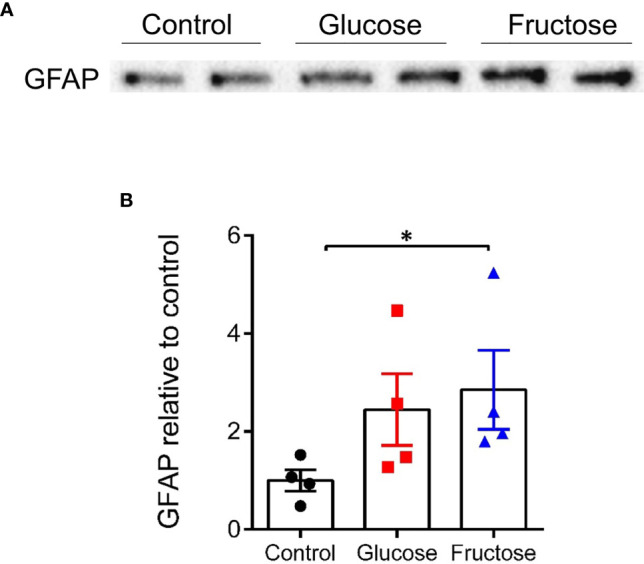
Protein expression level of GFAP in the cecum. **(A)** Representative images of Western blots for GFAP obtained from the load of 300 µg of cecal protein extract for each sample. All values are means ± SEM; *n* = 4/group. **(B)** Band intensity of the glucose and fructose group is expressed relative to the average intensity of the control group. Means were compared by Kruskal–Wallis test followed by Dunn multiple comparison test. * indicates significant differences among groups (*p < 0.05). GFAP, Glial fibrillary acidic protein.

Usually, pro-inflammatory cytokines such as IL13, TNFα, and IFNγ are associated with increased paracellular permeability (22, 57). Yet, only glucose, but not fructose increased the permeability in the cecum. To clarify this surprising observation, we investigated the impact of fructose and glucose on the EGCs of the ENS, which has been described as contributing to the maintenance of epithelial barrier function (36). GFAP protein levels were quantified in full thickness preparation from cecum segment. GFAP protein expression level significantly increased in cecum of mice drinking fructose when compared to the cecum of control mice (**Figures 4A, B**), suggesting active gliosis in response to fructose.

### In Pro-Inflammatory Context, Intestinal Permeability Changes in Response to Fructose Are Less Pronounced Than With Glucose

**Figure 5:**
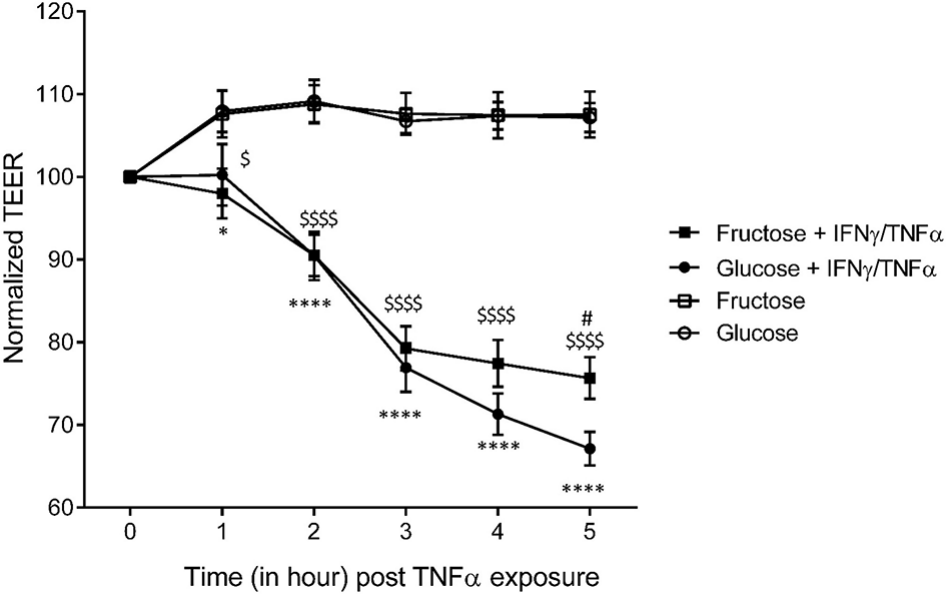
The effects of glucose and fructose on barrier function in response to cytokines in Caco-2 cells. Caco-2BBe cells were differentiated on transwells for 17 days after confluence until reaching stable TEER. Cells were cultured for 24 h in the presence of glucose (4.5 g/L)- or fructose (4.5 g/L)-enriched DMEM before exposure to cytokines. The cells were treated, in the presence of glucose or fructose, with 10 ng/ml IFNγ in the basal chamber for 24 h prior to 50 ng/ml TNFα treatment. The TEER was measured for 5 h following introduction of TNFα (= T0). All values are means ± SEM; *n* = 8/group. For each sample, TEER was normalized to values at T0. At each time point, means of normalized data were compared by Kruskal–Wallis test followed by Dunn multiple comparison test. * indicates a significant difference between Glucose+IFNγ/TNFα and Glucose groups (*p < 0.05; ****p < 0.0001), ^$^ indicates a significant difference between Fructose+IFNγ/TNFα and Fructose groups (^$^ *p* < 0.05; ^$$$$$^ *p* < 0.0001), and ^#^ indicates a significant difference between Fructose+IFNγ/TNFα and Glucose+IFNγ/TNFα groups (^#^ *p* < 0.05).

To investigate further the differential effects of glucose and fructose on intestinal permeability and to determine whether the two monosaccharides act directly on enterocytes, we used the Caco-2 cell line since these cells grow as polarized monolayers and express sugar transporters at the apical membrane (58). They have been extensively used as a model to study TJ regulation in response to pro-inflammatory cytokines (22, 59, 60). The permeability of tight junctions was measured by monitoring TEER. After differentiation of the Caco-2, enterocyte monolayers were cultured in the presence of 25 mM glucose or fructose for 48 h. Then, the TEER was measured in response to IFNγ priming followed by TNFα treatment. In the absence of pro-inflammatory challenge, 25 mM fructose did not affect the TEER when compared to 25 mM glucose (**Figure S2**). In both glucose and fructose culture conditions, TNFα/IFNγ caused a time-dependent decrease in Caco-2 TEER (**Figure 5**). However, when compared to glucose, fructose significantly attenuated the TEER decrease in response to cytokine stimulation after 5 h. The lack of effect of TNFα/IFNγ treatment on TEER when the glucose or fructose concentrations were reduced to 1 mM suggests that the sugar metabolism is a key factor enabling pro-inflammatory cytokines to reduce TEER (**Figure S2**).

### Glucose and Fructose Changed the Gut Microbiota Composition

**Figure 6:**
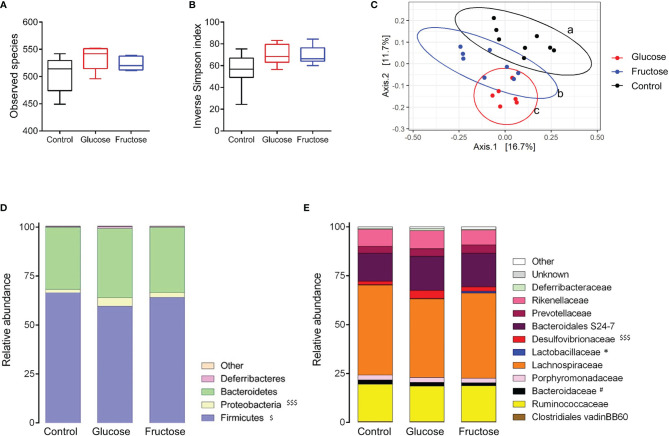
Impact of glucose and fructose on α- and β-diversity of bacterial cecum content. Analysis was based on 16S rDNA sequencing (region V3–V4) of the cecum content collected from control, glucose or fructose mice (*n* = 6–8/group). **(A)** Observed species richness and **(B)** Inverse Simpson Index as indicators of α-diversity. **(C)** Principal coordinates analysis (PCoA) of Bray-Curtis compositional dissimilarity at the operational taxonomic unit (OTU) level. Each dot represents of one mice. ^a, b, c^ indicate a significant difference between the groups. Average relative abundance at the **(D)** phylum and **(E)** family levels in caecum content of each group. For figures D and E, the average of each group is represented along the X-axis and the Y-axis refers to relative normalized abundances. Αlpha-diversity, phylum and family relative abundance data were compared using Kruskal-Wallis test followed by Dunn multiple comparison test. ^$^ indicate significantly different phyla or family abundances between Glucose and Control (with ^$^p < 0.05, ^$$$^ p<0.001), ^#^ indicate significantly different family abundances between Fructose and Control (with ^#^p <0.05) and * indicate significantly different family abundances between Fructose and Glucose (with *p < 0.05).

Several studies have reported that diet-induced changes in gut microbiota composition contribute to the intestinal inflammation and alteration of the gut permeability (37). Therefore, we investigated the effects of glucose- and fructose-drinking treatment on the cecal ecosystem composition using microbial 16S rRNA sequencing. The α-diversity was measured at the OTU level using the richness (observed species) and inverse Simpson index. Both parameters showed no significant change in richness and α-diversity of the microbiota in response to glucose or fructose intake (**Figures 6A, B**). However, PCoA of Bray–Curtis compositional dissimilarity between cecum samples from each group showed a significant separation between the communities in response to glucose and fructose when compared to the control group (*p* = 0.003 and *p* = 0.004, respectively) as well as between glucose and fructose (*p* = 0.007) (**Figure 6C**). Glucose-drinking mice demonstrated a more significant shift in cecal microbial composition than fructose when compared to the control mice. Firmicutes relative abundance decreased in the glucose group when compared to control while the relative abundance of Proteobacteria increased significantly in the cecal community of the glucose group when compared to control (**Figure 6D**). At a lower taxonomic level, within the Proteobacteria phylum, the relative abundance of *Desulfovibrionaceae* significantly increased in the cecal content of the glucose group when compared to control groups (**Figure 6E**). Within the Firmicutes phylum, the fructose intake increased the relative abundance of *Lactobacillaceae* when compared to the glucose group. Within the Bacteroidetes phylum, fructose modestly decreased the relative abundance of *Bacteroidaceae* when compared to the control group. The increase in *Desulfovibrionaceae* indicates that glucose consumption modified microbiota composition towards pro-inflammatory profile while the higher abundance in *Lactobacillaceae* suggests that beneficial microbiota environment was associated with fructose chronic intake.

**Figure 7:**
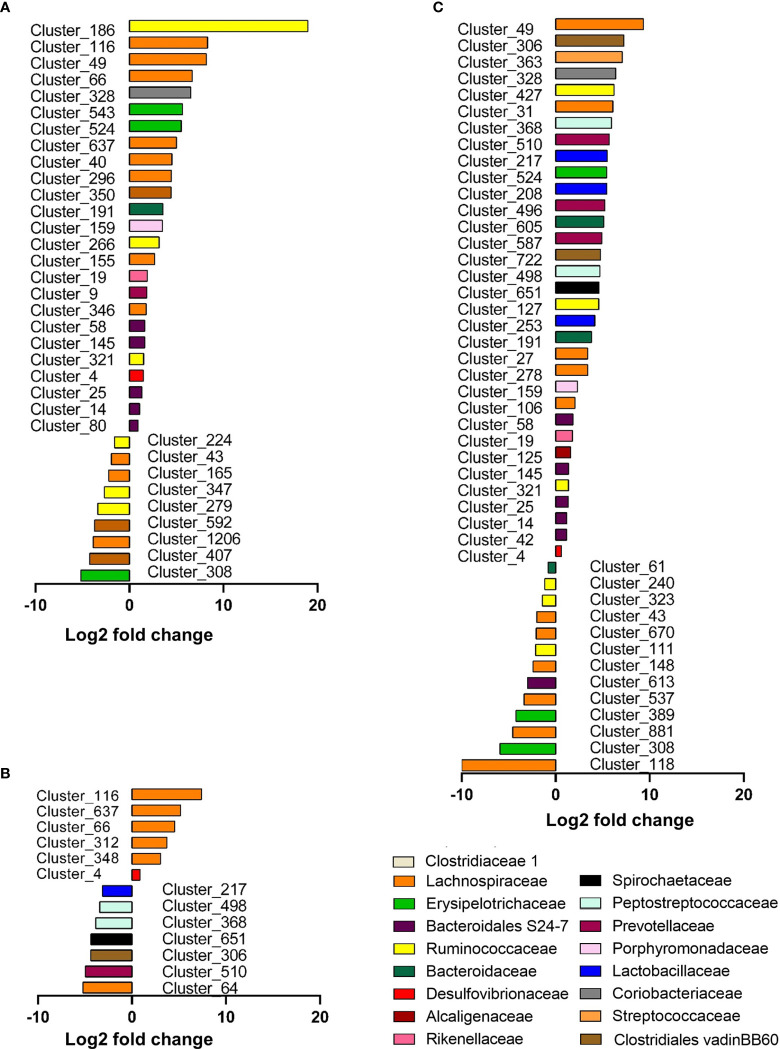
Graphic representation of differentially abundant OTUs between Glucose and Control groups **(A)**, Glucose and Fructose groups **(B)**, and Fructose and Control groups **(C)**. Only OTUs (named Cluster) with adjusted values <0.05, estimated fold change >1.5 or <1/1.5, and relative abundance >0.1% in at least half the samples were considered and included in the plots. Each OTU is represented by a bar colored according to its taxonomic classification at the family level. Taxonomy at the genus or species level is also indicated, when available, in **Supplementary Table S2**. A logarithmic scale (log-2) was used for the *x*-axis.

Differential abundance analysis identified 34 significant (*p* < 0.05) species or clusters that were positively (25 clusters) or negatively (9 clusters) differentially abundant between glucose and control groups (**Figure 7A**). Among them, 7 clusters exhibited a fold change > 32 and 11 of them belong to *Lachnospiraceae* family. This differential analysis also highlighted cluster_4 belonging to the *Desulfovibrio* genus. This cluster related to the most abundant species identified by DESeq2 (base mean = 719, **Table S2**), suggesting that the increase in *Desulfovibrionaceae* abundance in response to glucose identified previously (and confirmed by qRT-PCR) (**Figure S3**) was likely related to the increase in cluster_4 abundance (**Figures 7A, B**). Similarly, 46 species or clusters were positively (33 clusters) or negatively (13 clusters) differentially abundant between fructose and control groups and only 15 of them were similar to those identified as differentially abundant in response to glucose (**Figure 7C**). Among the species showing increased differential abundance in response to fructose, three species (cluster_253, cluster_208, and cluster_217) belong to the *Lactobacillus* family, which appears as a signature of the fructose intake (**Figures 7B, C**). Six species were positively (among which five species belong to *Lachnospiracae* family and one, cluster_4, to *Desulfovibrionaceae* family) and seven were negatively (among which the clutser_217 was from *Lactobacillaceae* family) differentially abundant between glucose and fructose groups (**Figure 7B**).

When ingested as liquid, glucose and fructose are not fully absorbed by the enterocytes and reach the lumen of the lower intestine (37, 61). *In vivo*, changes in the abundance of specific bacterial species under glucose or fructose suggest different abilities of the bacteria to use directly these two monosaccharides to promote their growth. We tested this hypothesis for the bacteria that showed differential abundance *in vivo* and that can be grown in culture by assessing their growth *in vitro* in the presence of either glucose or fructose. These bacteria were (1) either representative of bacteria that had an increased relative abundance in response to both glucose and fructose intake in comparison to the control group (cluster_191 identified as *B. vulgatus*, **Table S2**), (2) bacteria that displayed an enrichment specifically in response to fructose (*L. johnsonii* characteristic of *Lactobacillus* genus and corresponding to cluster_253), or (3) bacteria that displayed an enrichment specifically in response to glucose (as *D. vulgaris* characteristic of *Desulfovibrio* genus and corresponding likely to cluster_4). *B. vulgatus*, *L. johnsonii*, and *D. vulgaris* were all able to grow on either glucose- or fructose-containing media (**Figure S4**) and for all three species, growth was promoted by glucose in microbial culture media when compared to fructose.

### Correlation Between Gene Expression of Inflammatory Markers and Gut Microbiota

**Figure 8:**
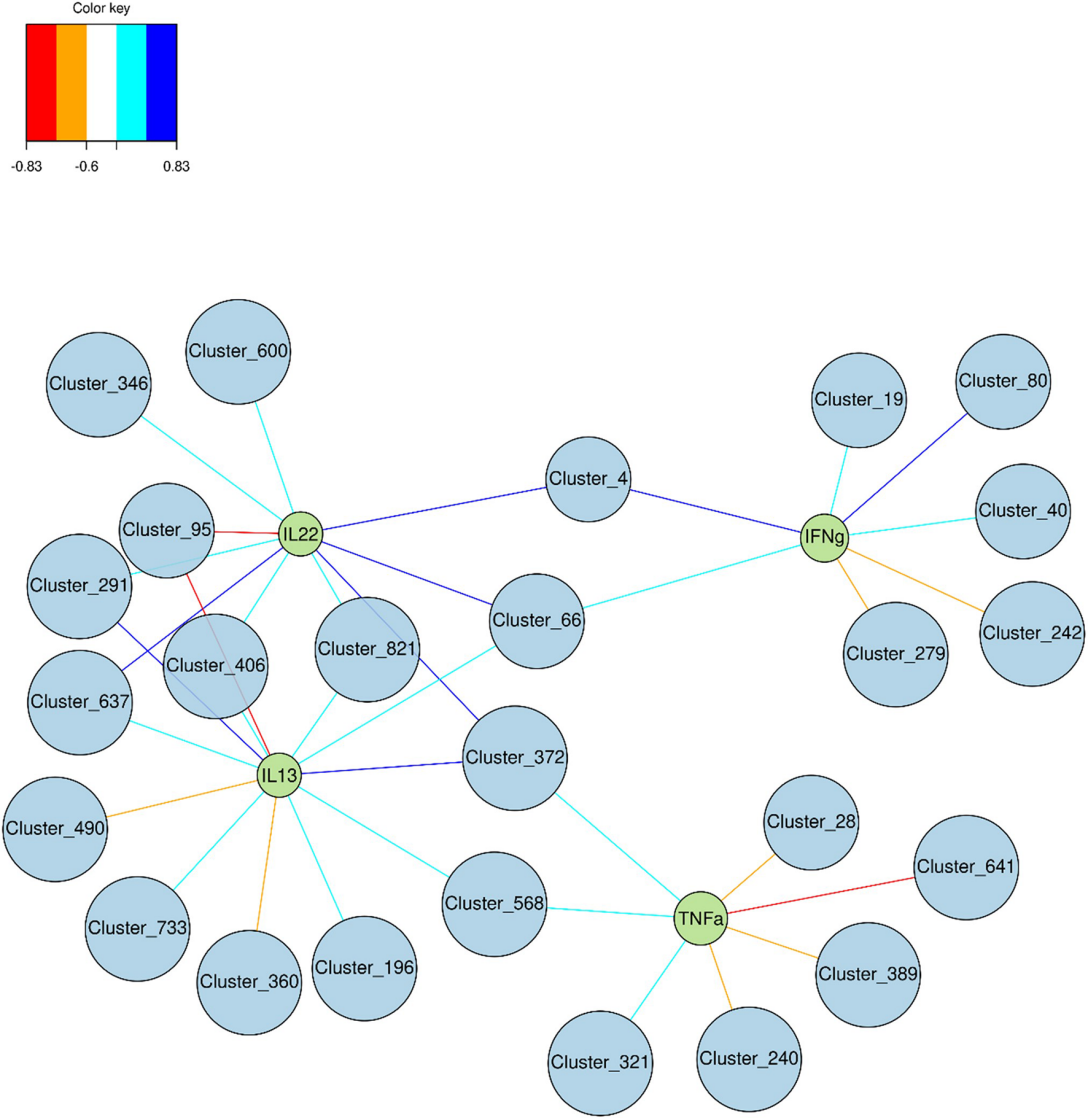
Graphic representation of the correlation between cytokine expression levels and relative abundance of bacterial species. Discriminant Clusters (blue circles and cluster ID) and cytokines (green circles and names) were positively (light and dark blue) or negatively (orange and red) connected.

Multi-group sPLS-DA was performed to identify the OTU that discriminated for components 1 and 2 between glucose, fructose, and control groups (**Figure S5**). Using a cutoff of ≥0.6, 25 bacterial species (clusters) were correlated with four pro-inflammatory cytokines’ (IL22, IL13, TNFα, and IFNγ) gene expression levels (**Figure 8**). Two clusters belonging to the *Clostridiales* order were positively linked to three cytokines: cluster_66 (*Lachnospiraceae* family) correlated to IL22, IFNγ, and IL13 and cluster_372 (*Ruminococcaceae* family) correlated with IL22, TNFα, and IL13. Four other clusters, all members of the *Clostridiales* order, were correlating positively with IL22 and IL13 (cluster_291, cluster_637, cluster_406, and cluster_821) while cluster_95 (*Oscillibacter* genus) was strongly negatively correlated to those. Cluster_4 (*Desulfovibrio* genus) was linked with Il22 and IFNγ while cluster_568 (*Clostridiales vadinBB60* family) was associated with TFNα and IL13. In addition to cluster_95, two other members of the *Bacteroidales S24-7 group* were negatively correlated to IL13 (cluster_490 and _360). Four clusters were also negatively associated to TNFα, among which two, cluster_240 (*Ruminococcaceae* family) and cluster_389 (*Lachnospiraceae* family), display a negative fold change in response to fructose intake (**Figure 7C**). Interestingly cluster_4, cluster_66, cluster_637, cluster_40 (*Lachnospiraceae* family), and cluster_80 (*Bacteroidales S24-7 group* family) were among the bacterial species significantly upregulated in response to glucose (**Figures 7A, B**), highlighting the potential role of glucose-induced gut microbiota changes in the development of inflammation in the epithelium of the lower GI tract.

## Discussion

In the current study, we provide experimental evidence that chronic intake of enriched fructose beverage did not alter the paracellular permeability in the jejunum, cecum, and colon and this despite a clear increase in systemic and cecal inflammation in these mice. Data using Caco-2 cells revealed that fructose may counteract the increase in inflammation-induced permeability, strengthening this lack of association between fructose and alteration of the intestinal paracellular permeability. To the opposite glucose intake was associated with a clear increase in paracellular permeability in the jejunum and the cecum and a strong inflammation in the cecum likely resulting from changes in the microbiota composition.

Our investigation of the consequence of fructose on intestinal paracellular permeability was motivated by strong evidence from mice models fed with high amounts of fructose that fructose triggers the disruption of intestinal TJ protein expression, especially occludin, ZO1, and claudin2 (29, 32, 34, 62). In our current study, despite a minor decrease in *occludin* and *claudin2* expression in the cecum in response to fructose intake, these changes were not associated with the alteration of intestinal permeability. In the cecum, while fructose consumption is associated with an increase in expression of *Il13, Tnfα*, and *Ifnγ* in a range similar to what is observed in response to glucose intake, the paracellular permeability remains unchanged in the mice receiving fructose when compared to the control group and increased only in the cecum of the glucose mice. The ability of pro-inflammatory cytokines to regulate TJ protein expression or assembly and to modulate epithelial permeability has been well described (22, 57, 63, 64). IL13 is known to increase the paracellular permeability through claudin2 (57, 65) while TNFα and IFNγ required the activation of the MLCK pathway (66). TNFα and IFNγ stimulate MLCK mRNA transcription, protein expression and activity that in turn activate the phosphorylation of the myosin light chain (MLC). The phosphorylated form of MLC results in the contraction of peri-junctional actinomyosin filaments and the TJ opening (22, 60, 66–68). This is coherent with the change in paracellular permeability observed in the cecum of the mice consuming glucose since the increase in TNFα and IFNγ was associated with a higher mRNA and protein expression of MLCK. However, even in a similar configuration, the mice consuming fructose failed to increase their intestinal epithelial paracellular permeability in the cecum. We also found *in vitro* that barrier permeability alteration induced by IFNγ/TNFα develops less rapidly when Caco-2 cells were cultured in the presence of fructose when compared to glucose. Thus, taken together, these data suggest that glucose, but not fructose, enhances the alteration intestinal paracellular permeability in a pro-inflammatory context. The mechanism underlying this finding remains to be clarified and may differ in the small intestine and in the cecum where the transport and metabolism efficacy of glucose and/or fructose are likely different. The phosphorylation of MLC by MLCK and subsequent actomyosin contraction is an ATP-dependent process (69). Unlike glucose-metabolizing enzymes, the metabolism of fructose results in ATP depletion due to the lack of control of the first steps of the fructose metabolism by KHK and aldolase (70). This depletion in ATP had been shown in fructose metabolizing tissues such as renal tubules (71, 72), liver (73), and intestine (74). Thus, in the enterocytes when the fructose transportation and metabolism is high, as in the small intestine exposed to fructose, the diminution of ATP levels may prevent ATP-dependent actomyosin contraction process. The sugar metabolism appears necessary for pro-inflammatory cytokine-induced permeability changes. Therefore, in the cecum, a lower absorption and metabolism of fructose than glucose by the epithelial cells may also contribute to reduce the impact of pro-inflammatory cytokines on intestinal permeability in that specific region.

Other factors known to participate in the regulation of the intestinal permeability may be involved in the weakening effects of inflammation on paracellular permeability in the presence of fructose *in vivo*. In the past few years, several studies have demonstrated that EGCs are involved in maintaining gut homeostasis (75). GFAP is a marker of the enteric glia (76, 77) and intestinal increase in GFAP protein expression has been reported to be a sign of reactive gliosis (78). Active gliosis has been observed in the gut of patients with inflammatory bowel disease (79, 80) and *in vitro* TFNα induced increase in GFAP level in EGC (77). Since ablation of GFAP-positive EGC resulted in severe gut inflammation (3, 79, 81), it had been proposed that increase in GFAP and reactive gliosis preserved the intestinal epithelium integrity, even if a recent study challenged this hypothesis (82). In our study, fructose-induced GFAP may be a sign of active gliosis, which one deployed its protective effects on the integrity of the intestinal epithelium *in vivo* and limits the deleterious effects of fructose-induced inflammation on intestinal permeability *in vivo*.

Previous studies showed that chronic intake of high level of fructose (about 65%) or of fructose + high fat diet was associated with a significant increase in intestinal permeability (29, 34) while intake of a moderate amount of fructose was not (83). Chronic intake of high level of fructose is often associated with an increased glycemia. Recently, hyperglycemia was demonstrated as a major trigger of intestinal permeability increase (28). This glycemia-induced increase in intestinal permeability may explain the dissimilar effects of fructose on intestinal permeability obtained in our study and the one observed by others. More importantly, this hypothesis suggests that the increase in gut permeability does not precede the low-grade inflammation and glucose intolerance often observed in response to dietary fructose. On the contrary, the alteration of the gut permeability could be one of the consequences of the low-grade inflammation and glucose intolerance as published recently (28).

Interestingly glucose and fructose drink intake led to different intestinal inflammation profile in the various regions of the GI tract. Recent studies revealed the metabolic reprogramming of immune cells in response to sugars (84, 85). Fructose and glucose absorption take place mainly in the proximal intestine (duodenum and jejunum). Their trans-epithelial transport is ensured at the apical membrane of the enterocytes by the GLUT5 and SGLT1 transporters, respectively, and by GLUT2 at the basolateral side (86). Therefore, after ingestion of food and drinks enriched in sugar, the highest concentrations of glucose and fructose are found in the jejunum (87, 88). Regardless of those high concentrations, fructose and glucose had almost no effect on the cytokine expression levels in the jejunum. Only glucose intake was associated with an increase in *Il1β* mRNA levels suggesting the activation of the macrophages. Conversely, a robust inflammation was observed in the cecum in response to both glucose and fructose intake. Based on the profile of cytokine expression, glucose and fructose intake may have activated the immune response involving the lymphocytes Type 1 helper (Th1) or the innate lymphoid cells (ILC1) producing TNFα and IFNγ in the cecum (89). The lymphocytes Type 2 helper (Th2) or the ILC2 producing Il13 may also be involved. In addition, glucose but not fructose may activate the lymphocytes Type 17 helper (Th17) producing IL22. The increase in *Il10* expression suggested also the possible activation of macrophages and regulatory T cells (Treg) in the lamina propria in the response to glucose and fructose intake. However, the direct role of the sugars or the indirect role of the microbiota in the response observed in our study remain to be demonstrated. Intestinal transport of fructose is poorly efficient when compared to glucose and a substantial fraction of fructose overspills in the distal parts of the GI where it is metabolized by the bacteria (37, 48). Recent studies clearly demonstrate the link between diet and microbiota in the enhancement of the gut inflammation, and more specifically, between microbiota and colitis suspectibility in reponse to glucose (83, 90). We propose that the adaptation of the cecal ecosystem to glucose and fructose intake, shown by the change in β-diversity, plays a role in the onset of cecal inflammation. Among the bacteria tested *in vitro* in the current study, glucose and even fructose can support bacterial growth. The most prominent family affected by glucose, and to a lesser extent by fructose, was the *Desulfovibrionaceae.* Interestingly, *Desulfovibrionaceae* are endotoxin producers (91) and have been linked to glucose tolerance in response to Western diet intake in mice (92) and to obesity in human (93). Moreover, *Desulfovibrionaceae* are sulfate-reducing bacteria that generate hydrogen sulfide (H_2_S) (94) and the abundance of H_2_S-producing bacteria has been correlated to intestinal inflammation, activation of immune cells, and alteration of permeability (95–97). Intake of glucose was also characterized by an enrichment of *Lachnospiraceae_NK4A136 group*, which has been reported to increase in rodent model of obesity and colitis (98, 99). *Lachnospiraceae* produce butyrate, a short-chain fatty acid known for its beneficial effects on host health. However, *Lachnospiraceae* increased abundance has been also positively associated with several diseases (100). Interestingly, the response of intestinal epithelial cells to butyrate depends on the presence or absence of other energetic substrates: while butyrate promotes cell growth in the absence of glucose, it stimulates apoptosis at similar concentration when glucose is also available (101). This latter situation may resemble what occurred in the glucose fed mice in our study. Finally, in agreement with one earlier study (48) but in contrast with another (83), we found that mice consuming fructose displayed augmentation of *Lactobacillaceae*, which have been shown to exert health benefits including enhancement of epithelial barrier function and modulation of immune responses in rodent and human (100). The Lactobacilli may protect the epithelial barrier by inducing mucin secretion (102), stabilizing the tight junctions (103), reducing the apoptosis of epithelial cells (104) or supporting the proliferation of intestinal stem cells (105). Therefore, in our study, fructose-induced *Lactobacillaceae* may prevent alteration of the gut barrier function in response to fructose intake.

This study provides strong evidence that physiological amounts of fructose do not alter the intestinal paracellular permeability in any of the regions of the GI tract despite an increase in cecal epithelium inflammation and in circulating levels of IL6. Our data strongly suggest that alteration of intestinal permeability does not precede but follows the onset of metabolic outcome (low-grade inflammation and hyperglycemia) associated with chronic fructose consumption. We have also demonstrated that despite being absorbed in the small intestine, glucose intake has major harmful effects on the intestinal paracellular permeability of the cecum where it may interact with the microbiota. In particular, glucose-induced increased abundance of bacteria of *Desulfovibrionaceae* and *Lachnospiraceae_NK4A136 group* families might participate to the deleterious outcome of high glucose consumption on the intestinal epithelium.

## Data Availability Statement

The data presented in the study are deposited in Data INRAE Omic dataverse repository, https://doi.org/10.15454/3LBOIS.

## Ethics Statement

The animal study was reviewed and approved by the Ethics Committee in Animal Experiment of INRA Jouy-en-Josas (Comethea, registration number: APAFIS#1620-2015102618572930v2).

## Author Contributions

The authors’ responsibilities were as follows: XZ, CH, and VD designed the research. XZ, FB-C, CH, and VD conducted the research. XZ, CH, MMo, MMa, and VD analyzed the data. XZ and VD wrote the manuscript. XZ, CH, NL, and VD contributed to the discussion. All authors contributed to the article and approved the submitted version.

## Conflict of Interest

The authors declare that the research was conducted in the absence of any commercial or financial relationships that could be construed as a potential conflict of interest.

## Publisher’s Note

All claims expressed in this article are solely those of the authors and do not necessarily represent those of their affiliated organizations, or those of the publisher, the editors and the reviewers. Any product that may be evaluated in this article, or claim that may be made by its manufacturer, is not guaranteed or endorsed by the publisher.

## Abbreviations

DMEM, Dulbecco’s modified Eagle’s medium; ECL-HRP,Enhanced chemiluminescent horseradish peroxidase; EGC, Enteric glial cells; ENS, Enteric nervous system; FBS, Fetal bovine serum; FITC-SA, Fluorescein isothiocyanate-Sulfuric acid; GAPDH, Glyceraldehyde 3-phosphate dehydrogenase; GFAP, Glial fibrillary acidic protein; GI, Gastrointestinal; GLUT2, Facilitated glucose transporter 2; GLUT5, Facilitated glucose/fructose transporter 5; IFN-g, Interferon gamma; IL1b, Interleukin 1 beta; IL10, Interleukin 10; IL6, Interleukin 6; IL13, Interleukin 13; IL22, Interleukin 22; ILC1 or 2, Group 1 or 2 innate lymphoid cells; JAMs, Junctional adhesion molecules; MLC, Myosin light chain; MLCK: Myosin light chain kinase; OGTT, Oral glucose tolerance test; OTU: Operational taxonomic units; PCoA, Principal coordinates analysis; SGLT1, Apical sodium/glucose cotransporter; sPLS-DA: Sparse partial least squaresdiscriminant analysis; TEER, Transepithelial electrical resistance; Th1, Lymphocytes type 1 helper; Th2, Lymphocytes type 2 helper; Th17, Lymphocytes type 17 helper; TJ, Tight junction; TNF-a, Tumor necrosis factor alpha; Treg, Regulatory T cells; ZO, Zonula occludens.
