# Supplementary material for: Glucose but Not Fructose Alters the Intestinal Paracellular Permeability in Association With Gut Inflammation and Dysbiosis in Mice
Source: Front Immunol. 2021 Dec 27;12:742584. doi: 10.3389/fimmu.2021.742584 (PMC8744209; doi:10.3389/fimmu.2021.742584)
Supplement: Supplementary file 1 [file DataSheet_1.pdf]

Supplementary information

Table S1: Pirmer used for SYBR green Quantitative Real Time-PCR

Name (Gene)	Gene Bank accession n°	Primers
	**Primers for SYBR Green**	
β Actin *(beta-actin)*	NM_007393	Forward: 5’-CTAAGGCCAACCGTGAAAAG-3’
		Reverse: 5’-ACCAGAGGCATACAGGGACA-3’
tight junction protein 1 (*Tjp1 or ZO1*)	NM_009386	Forward: 5’- GATCATTCCACGCAGTCTCC -3’
		Reverse: 5’- GGCCCCAGGTTTAGACATTC -3’
Claudin2 (*Cldn2*)	NM_016675	Forward: 5’- GTAGCCGGAGTCATCCTTTG -3’
		Reverse: 5’- GGCCTGGTAGCCATCATAGT -3’
Occludin (*Ocln*)	NM_008756	Forward: 5’- GCGATCATACCCAGAGTCTTTC -3’
		Reverse: 5’- TGCCTGAAGTCATCCACACT -3’
Myosin light polypeptide kinase (*Mlck*)	NM_139300	Forward: 5’- ATAGCCTTGGCCAGGTGTC -3’
		Reverse: 5’- GCTGTCTGTGCCAAGTTCG -3’
Interleukin 1 beta (*Il1β*)	NM_008361	Forward: 5’- AGTTGACGGACCCCAAAAG -3’
		Reverse: 5’- AGCTGGATGCTCTCATCAGG -3’
Tumor necrosis factor (*Tnf*)	NM_013693	Forward: 5’-CTGTAGCCCACGTCGTAGC-3’
		Reverse: 5’-TTGAGATCCATGCCGTTG-3’
Interleukin 13 (*Il13*)	NM_008355	Forward: 5’-ACCCAGAGGATATTGCATGG-3’
		Reverse: 5’-TGGGCTACTTCGATTTTGGT-3’
Interferon gamma (*Ifnγ*)	NM_008337	Forward: 5’-ATCTGGAGGAACTGGCAAAA-3’
		Reverse: 5’-TTCAAGACTTCAAAGAGTCTGAGGTA-3’
Interleukin 10 (*Il10*)	NM_010548	Forward: 5’- CAGAGCCACATGCTCCTAGA -3’
		Reverse: 5’- TGTCCAGCTGGTCCTTTGTT -3’
Interleukin 22 (*Il22*)	NM_016971	Forward: 5’- TGACGACCAGAACATCCAGA -3’
		Reverse: 5’- AATCGCCTTGATCTCTCCAC -3’
GLUT5 *(Slc2a5*)	NM_019741	Forward: 5’- TGCAGAGCAACGATGGAGAAA-3’
		Reverse: 5’- ACAGCAGCGTCAGGGTGAAG-3’
GLUT2 (*Slc2a2*)	NM_031197	Forward: 5’- TGTGATCCAGTGAGTCTCCAA-3’
		Reverse: 5’- GGCGCACATCTATAATGCTCT-3’
SGLT1 (*Slc5a1*)	NM_019810	Forward: 5’- CATCAGCGTCATCACCATCT-3’
		Reverse: 5’- TCCAACACAAACGGTACAGG-3’

Table S2 : Data resulting from Desq2 comparison of bacterial specie abundance between glucose and control groups ; fructose and control groups or glucose and fructose groups.

Desq2 comparison glucose/control

baseMean	log2FoldChange	padj	cluster ID	phylum	class	order	family	genus	species
11,94	-5,19	2,74E-03	Cluster_308	Firmicutes	Erysipelotrichia	Erysipelotrichales	Erysipelotrichaceae	Unknown	Unknown
6,04	-4,23	2,12E-02	Cluster_407	Firmicutes	Clostridia	Clostridiales	Clostridiales vadinBB60 group	Unknown	Unknown
1,94	-3,86	2,94E-02	Cluster_1206	Firmicutes	Clostridia	Clostridiales	Lachnospiraceae	Lachnospiraceae NK4A136 group	Unknown
2,00	-3,71	1,30E-02	Cluster_592	Firmicutes	Clostridia	Clostridiales	Clostridiales vadinBB60 group	Unknown	Unknown
12,94	-3,38	8,47E-03	Cluster_279	Firmicutes	Clostridia	Clostridiales	Ruminococcaceae	Ruminiclostridium	Unknown
8,63	-2,67	1,68E-02	Cluster_347	Firmicutes	Clostridia	Clostridiales	Ruminococcaceae	Ruminococcaceae UCG-014	Unknown
37,59	-2,21	3,08E-02	Cluster_165	Firmicutes	Clostridia	Clostridiales	Lachnospiraceae	Lachnospiraceae UCG-001	Unknown
201,37	-1,93	7,20E-03	Cluster_43	Firmicutes	Clostridia	Clostridiales	Lachnospiraceae	Lachnospiraceae NK4A136 group	Unknown
19,92	-1,59	2,94E-02	Cluster_224	Firmicutes	Clostridia	Clostridiales	Ruminococcaceae	Ruminiclostridium 5	Unknown
95,74	0,90	3,51E-02	Cluster_80	Bacteroidetes	Bacteroidia	Bacteroidales	Bacteroidales S24-7 group	Unknown	Unknown
312,21	1,08	1,58E-03	Cluster_14	Bacteroidetes	Bacteroidia	Bacteroidales	Bacteroidales S24-7 group	Unknown	Unknown
222,55	1,30	2,12E-02	Cluster_25	Bacteroidetes	Bacteroidia	Bacteroidales	Bacteroidales S24-7 group	Unknown	Unknown
719,32	1,47	3,19E-09	Cluster_4	Proteobacteria	Deltaproteobacteria	Desulfovibrionales	Desulfovibrionaceae	Desulfovibrio	Unknown
10,46	1,50	3,72E-02	Cluster_321	Firmicutes	Clostridia	Clostridiales	Ruminococcaceae	Unknown	Unknown
49,97	1,62	6,56E-03	Cluster_145	Bacteroidetes	Bacteroidia	Bacteroidales	Bacteroidales S24-7 group	Unknown	Unknown
139,76	1,63	5,07E-05	Cluster_58	Bacteroidetes	Bacteroidia	Bacteroidales	Bacteroidales S24-7 group	Unknown	Unknown
12,31	1,77	6,56E-03	Cluster_346	Firmicutes	Clostridia	Clostridiales	Lachnospiraceae	Unknown	Unknown
413,60	1,84	4,43E-02	Cluster_9	Bacteroidetes	Bacteroidia	Bacteroidales	Prevotellaceae	Alloprevotella	Unknown
267,51	1,88	3,54E-04	Cluster_19	Bacteroidetes	Bacteroidia	Bacteroidales	Rikenellaceae	Alistipes	Unknown
45,75	2,67	3,49E-02	Cluster_155	Firmicutes	Clostridia	Clostridiales	Lachnospiraceae	Lachnoclostridium	Unknown
20,53	3,16	6,20E-03	Cluster_266	Firmicutes	Clostridia	Clostridiales	Ruminococcaceae	Ruminiclostridium 9	Unknown
40,91	3,50	1,79E-04	Cluster_159	Bacteroidetes	Bacteroidia	Bacteroidales	Porphyromonadaceae	Parabacteroides	Parabacteroides goldsteinii
31,89	3,57	2,44E-04	Cluster_191	Bacteroidetes	Bacteroidia	Bacteroidales	Bacteroidaceae	Bacteroides	Bacteroides vulgatus
13,05	4,42	5,62E-04	Cluster_350	Firmicutes	Clostridia	Clostridiales	Clostridiales vadinBB60 group	Unknown	Unknown
13,04	4,46	2,94E-02	Cluster_296	Firmicutes	Clostridia	Clostridiales	Lachnospiraceae	Unknown	Unknown
178,94	4,52	6,36E-03	Cluster_40	Firmicutes	Clostridia	Clostridiales	Lachnospiraceae	Coprococcus 1	Unknown
1,86	4,99	1,21E-03	Cluster_637	Firmicutes	Clostridia	Clostridiales	Lachnospiraceae	Lachnospiraceae NK4A136 group	Unknown
4,64	5,51	3,51E-02	Cluster_524	Firmicutes	Erysipelotrichia	Erysipelotrichales	Erysipelotrichaceae	Allobaculum	Unknown
3,42	5,61	2,12E-02	Cluster_543	Firmicutes	Erysipelotrichia	Erysipelotrichales	Erysipelotrichaceae	Erysipelatoclostridium	Unknown
11,71	6,52	1,76E-03	Cluster_328	Actinobacteria	Coriobacteriia	Coriobacteriales	Coriobacteriaceae	Coriobacteriaceae UCG-002	Unknown
107,59	6,69	2,28E-09	Cluster_66	Firmicutes	Clostridia	Clostridiales	Lachnospiraceae	Lachnospiraceae NK4A136 group	Unknown
172,43	8,17	1,06E-05	Cluster_49	Firmicutes	Clostridia	Clostridiales	Lachnospiraceae	Lachnospiraceae NK4A136 group	Unknown
14,34	8,32	2,78E-03	Cluster_116	Firmicutes	Clostridia	Clostridiales	Lachnospiraceae	Unknown	Lachnospiraceae bacterium 3-1
30,37	18,97	3,77E-12	Cluster_186	Firmicutes	Clostridia	Clostridiales	Ruminococcaceae	[Eubacterium] coprostanoligenes group	Unknown

Desq2 comparison fructose/control

baseMean	log2FoldChange	padj	cluster ID	phylum	class	order	family	genus	species
53,00	-10,04	1,48E-03	Cluster_118	Clostridia	Clostridiales	Lachnospiraceae	Lachnospiraceae	Lachnospiraceae NK4A136 group	Unknown
11,94	-5,93	1,25E-04	Cluster_308	Erysipelotrichia	Erysipelotrichales	Erysipelotrichaceae	Erysipelotrichaceae	Unknown	Unknown
1,87	-4,57	2,92E-02	Cluster_881	Clostridia	Clostridiales	Lachnospiraceae	Lachnospiraceae	Lachnospiraceae NK4A136 group	Unknown
2,34	-4,24	4,29E-02	Cluster_537	Erysipelotrichia	Erysipelotrichales	Erysipelotrichaceae	Erysipelotrichaceae	Unknown	Unknown
6,47	-3,38	1,51E-02	Cluster_389	Clostridia	Clostridiales	Lachnospiraceae	Lachnospiraceae	Unknown	Unknown
2,43	-3,00	2,85E-02	Cluster_613	Bacteroidia	Bacteroidales	Bacteroidales S24-7 group	Bacteroidales S24-7 group	Unknown	Unknown
40,79	-2,41	2,70E-02	Cluster_148	Clostridia	Clostridiales	Lachnospiraceae	Lachnospiraceae	Unknown	Unknown
66,19	-2,14	4,61E-02	Cluster_111	Clostridia	Clostridiales	Ruminococcaceae	Ruminococcaceae	Unknown	Unknown
5,51	-2,09	2,79E-02	Cluster_670	Clostridia	Clostridiales	Lachnospiraceae	Lachnospiraceae	Lachnospiraceae NK4A136 group	Unknown
201,37	-2,03	8,90E-04	Cluster_43	Clostridia	Clostridiales	Lachnospiraceae	Lachnospiraceae	Lachnospiraceae NK4A136 group	Unknown
9,49	-1,42	2,84E-02	Cluster_323	Clostridia	Clostridiales	Ruminococcaceae	Ruminococcaceae	Ruminiclostridium 5	Unknown
27,22	-1,17	3,41E-02	Cluster_240	Clostridia	Clostridiales	Ruminococcaceae	Ruminococcaceae	Oscillibacter	Unknown
104,68	-0,80	4,72E-02	Cluster_61	Bacteroidia	Bacteroidales	Bacteroidaceae	Bacteroidaceae	Bacteroides	Unknown
719,32	0,61	3,64E-02	Cluster_4	Deltaproteobacteria	Desulfovibrionales	Desulfovibrionaceae	Desulfovibrionaceae	Desulfovibrio	Unknown
161,18	1,17	1,16E-05	Cluster_42	Bacteroidia	Bacteroidales	Bacteroidales S24-7 group	Bacteroidales S24-7 group	Unknown	Unknown
312,21	1,18	5,76E-05	Cluster_14	Bacteroidia	Bacteroidales	Bacteroidales S24-7 group	Bacteroidales S24-7 group	Unknown	Unknown
222,55	1,31	7,68E-03	Cluster_25	Bacteroidia	Bacteroidales	Bacteroidales S24-7 group	Bacteroidales S24-7 group	Unknown	Unknown
10,46	1,35	4,45E-02	Cluster_321	Clostridia	Clostridiales	Ruminococcaceae	Ruminococcaceae	Unknown	Unknown
49,97	1,36	1,51E-02	Cluster_145	Bacteroidia	Bacteroidales	Bacteroidales S24-7 group	Bacteroidales S24-7 group	Unknown	Unknown
67,49	1,58	3,59E-02	Cluster_125	Betaproteobacteria	Burkholderiales	Alcaligenaceae	Alcaligenaceae	Parasutterella	Unknown
267,51	1,77	1,61E-04	Cluster_19	Bacteroidia	Bacteroidales	Rikenellaceae		Rikenellaceae	Alistipes
139,76	1,82	1,88E-07	Cluster_58	Bacteroidia	Bacteroidales	Bacteroidales S24-7 group	Bacteroidales S24-7 group	Unknown	Unknown
94,69	2,03	1,06E-02	Cluster_106	Clostridia	Clostridiales	Lachnospiraceae	Lachnospiraceae	Unknown	Unknown
40,91	2,31	1,51E-02	Cluster_159	Bacteroidia	Bacteroidales	Porphyromonadaceae	Porphyromonadaceae	Parabacteroides	Parabacteroides goldsteinii
15,12	3,39	2,73E-03	Cluster_278	Clostridia	Clostridiales	Lachnospiraceae	Lachnospiraceae	Lachnospiraceae UCG-008	Unknown
204,32	3,39	3,41E-02	Cluster_27	Clostridia	Clostridiales	Lachnospiraceae	Lachnospiraceae	Unknown	Unknown
31,89	3,82	7,02E-06	Cluster_191	Bacteroidia	Bacteroidales	Bacteroidaceae	Bacteroidaceae	Bacteroides	Bacteroides vulgatus
23,76	4,16	3,28E-04	Cluster_253	Bacilli	Lactobacillales	Lactobacillaceae	Lactobacillaceae	Lactobacillus	Unknown
110,34	4,57	1,61E-03	Cluster_127	Clostridia	Clostridiales	Ruminococcaceae	Ruminococcaceae	Ruminococcaceae UCG-014	Unknown
1,94	4,61	5,28E-04	Cluster_651	Spirochaetes	Spirochaetales	Spirochaetaceae	Spirochaetaceae	Treponema 2	Unknown
3,63	4,68	2,61E-05	Cluster_498	Clostridia	Clostridiales	Peptostreptococcaceae	Peptostreptococcaceae	Romboutsia	Unknown
1,87	4,77	7,98E-03	Cluster_722	Clostridia	Clostridiales	Clostridiaceae 1	Clostridiaceae 1	Clostridium sensu stricto 1	Unknown
2,53	4,92	6,83E-03	Cluster_587	Bacteroidia	Bacteroidales	Prevotellaceae	Prevotellaceae	Prevotella 9	Unknown
2,21	5,11	1,51E-02	Cluster_605	Bacteroidia	Bacteroidales	Bacteroidaceae	Bacteroidaceae	Bacteroides	Unknown
3,11	5,21	5,23E-03	Cluster_496	Bacteroidia	Bacteroidales	p-2534-18B5 gut group	p-2534-18B5 gut group	Unknown	Unknown
7,76	5,42	2,70E-04	Cluster_208	Bacilli	Lactobacillales	Lactobacillaceae	Lactobacillaceae	Lactobacillus	Unknown
4,64	5,44	2,00E-02	Cluster_524	Erysipelotrichia	Erysipelotrichales	Erysipelotrichaceae	Erysipelotrichaceae	Allobaculum	Unknown
23,84	5,49	1,92E-07	Cluster_217	Bacilli	Lactobacillales	Lactobacillaceae	Lactobacillaceae	Lactobacillus	Unknown
4,10	5,70	1,61E-04	Cluster_510	Bacteroidia	Bacteroidales	Prevotellaceae	Prevotellaceae	Prevotella 9	Unknown
8,21	5,93	1,65E-06	Cluster_368	Clostridia	Clostridiales	Peptostreptococcaceae	Peptostreptococcaceae	Terrisporobacter	Unknown
17,59	6,07	3,62E-02	Cluster_31	Clostridia	Clostridiales	Lachnospiraceae	Lachnospiraceae	Lachnospiraceae NK4A136 group	Unknown
4,65	6,19	3,28E-04	Cluster_427	Clostridia	Clostridiales	Ruminococcaceae	Ruminococcaceae	Ruminococcaceae UCG-008	Unknown
11,71	6,38	5,88E-04	Cluster_328	Coriobacteriia	Coriobacteriales	Coriobacteriaceae	Coriobacteriaceae	Coriobacteriaceae UCG-002	Unknown
8,37	7,06	7,87E-04	Cluster_363	Bacilli	Lactobacillales	Streptococcaceae	Streptococcaceae	Streptococcus	Unknown
12,19	7,26	3,88E-06	Cluster_306	Clostridia	Clostridiales	Clostridiaceae 1	Clostridiaceae 1	Clostridium sensu stricto 1	Unknown
172,43	9,33	1,78E-08	Cluster_49	Clostridia	Clostridiales	Lachnospiraceae	Lachnospiraceae	Lachnospiraceae NK4A136 group	Unknown
30,37	23,02	1,10E-21	Cluster_186	Clostridia	Clostridiales	Ruminococcaceae	Ruminococcaceae	[Eubacterium] coprostanoligenes group	Unknown

Desq2 comparison glucose/fructose

baseMean	log2FoldChange	padj	cluster ID	phylum	class	order	family	genus	species
114,46	-5,21	0,03	Cluster_64	Firmicutes	Clostridia	Clostridiales	Lachnospiraceae	Lachnospiraceae NK4A136 group	Unknown
4,10	-4,97	0,02	Cluster_510	Bacteroidetes	Bacteroidia	Bacteroidales	Prevotellaceae	Prevotella 9	Unknown
12,19	-4,38	0,03	Cluster_306	Firmicutes	Clostridia	Clostridiales	Clostridiaceae 1	Clostridium sensu stricto 1	Unknown
1,94	-4,36	0,02	Cluster_651	Spirochaetae	Spirochaetes	Spirochaetales	Spirochaetaceae	Treponema 2	Unknown
8,21	-3,86	0,01	Cluster_368	Firmicutes	Clostridia	Clostridiales	Peptostreptococcaceae	Terrisporobacter	Unknown
3,63	-3,43	0,01	Cluster_498	Firmicutes	Clostridia	Clostridiales	Peptostreptococcaceae	Romboutsia	Unknown
23,84	-3,12	0,03	Cluster_217	Firmicutes	Bacilli	Lactobacillales	Lactobacillaceae	Lactobacillus	Unknown
719,32	0,85	0,01	Cluster_4	Proteobacteria	Deltaproteobacteria	Desulfovibrionales	Desulfovibrionaceae	Desulfovibrio	Unknown
8,97	3,02	0,02	Cluster_348	Firmicutes	Clostridia	Clostridiales	Lachnospiraceae	Unknown	Unknown
11,51	3,71	0,02	Cluster_312	Firmicutes	Clostridia	Clostridiales	Lachnospiraceae	Unknown	Unknown
107,59	4,52	0,001	Cluster_66	Firmicutes	Clostridia	Clostridiales	Lachnospiraceae	Lachnospiraceae NK4A136 group	Unknown
1,86	5,15	0,003	Cluster_637	Firmicutes	Clostridia	Clostridiales	Lachnospiraceae	Lachnospiraceae NK4A136 group	Unknown
14,34	7,40	0,02	Cluster_116	Firmicutes	Clostridia	Clostridiale	Lachnospiraceae	Unknown	Lachnospiraceae bacterium 3-1
