# Supplementary material for: Glucose but Not Fructose Alters the Intestinal Paracellular Permeability in Association With Gut Inflammation and Dysbiosis in Mice
Source: Front Immunol. 2021 Dec 27;12:742584. doi: 10.3389/fimmu.2021.742584 (PMC8744209; doi:10.3389/fimmu.2021.742584)
Supplement: Supplementary file 2 [file Presentation_1.pptx]

## Slide 1
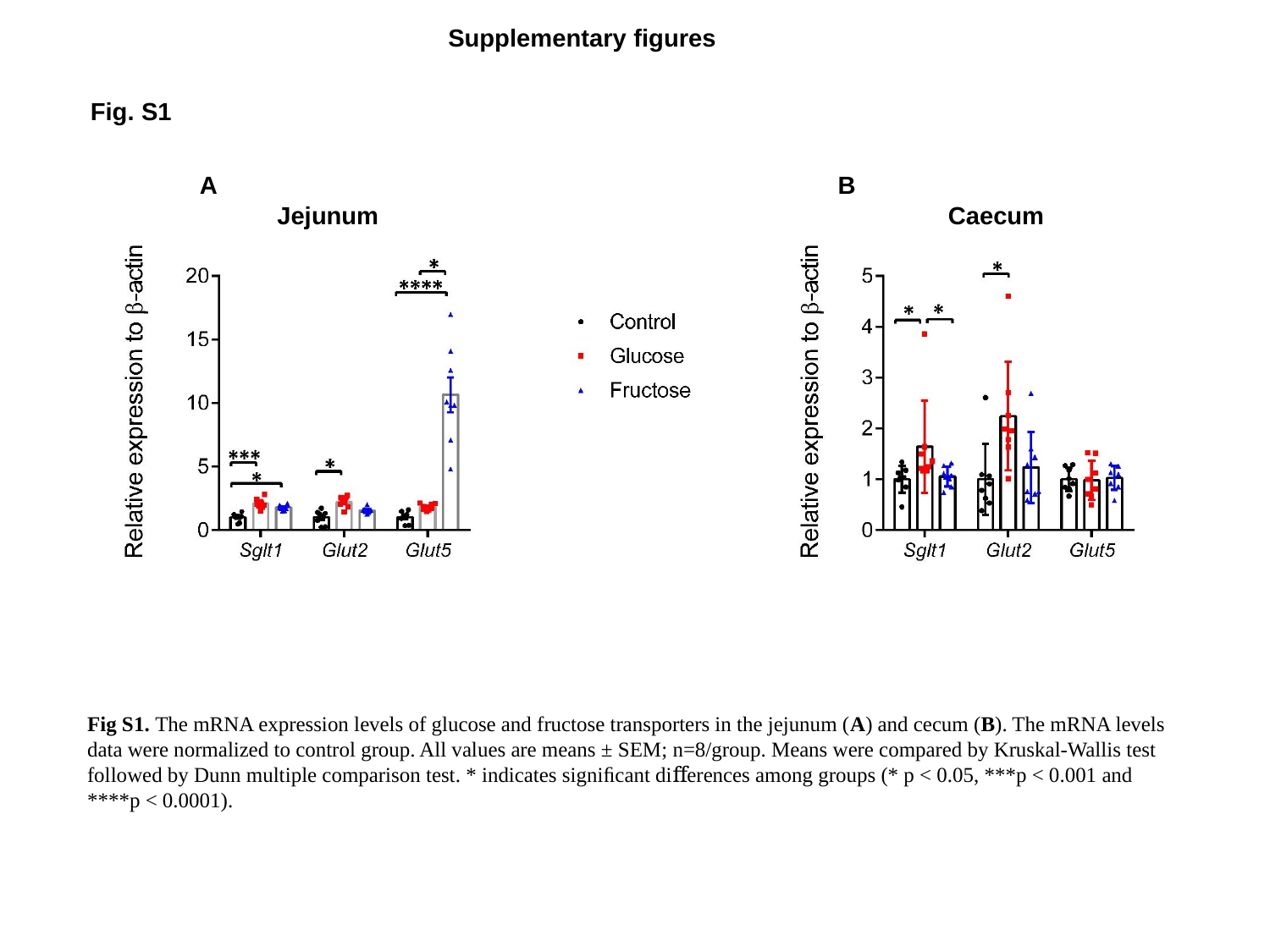

Supplementary figures
Fig. S1
 A
B
Jejunum
Caecum
Fig S1. The mRNA expression levels of glucose and fructose transporters in the jejunum (A) and cecum (B). The mRNA levels data were normalized to control group. All values are means ± SEM; n=8/group. Means were compared by Kruskal-Wallis test followed by Dunn multiple comparison test. * indicates signiﬁcant diﬀerences among groups (* p < 0.05, ***p < 0.001 and ****p < 0.0001).

## Slide 2
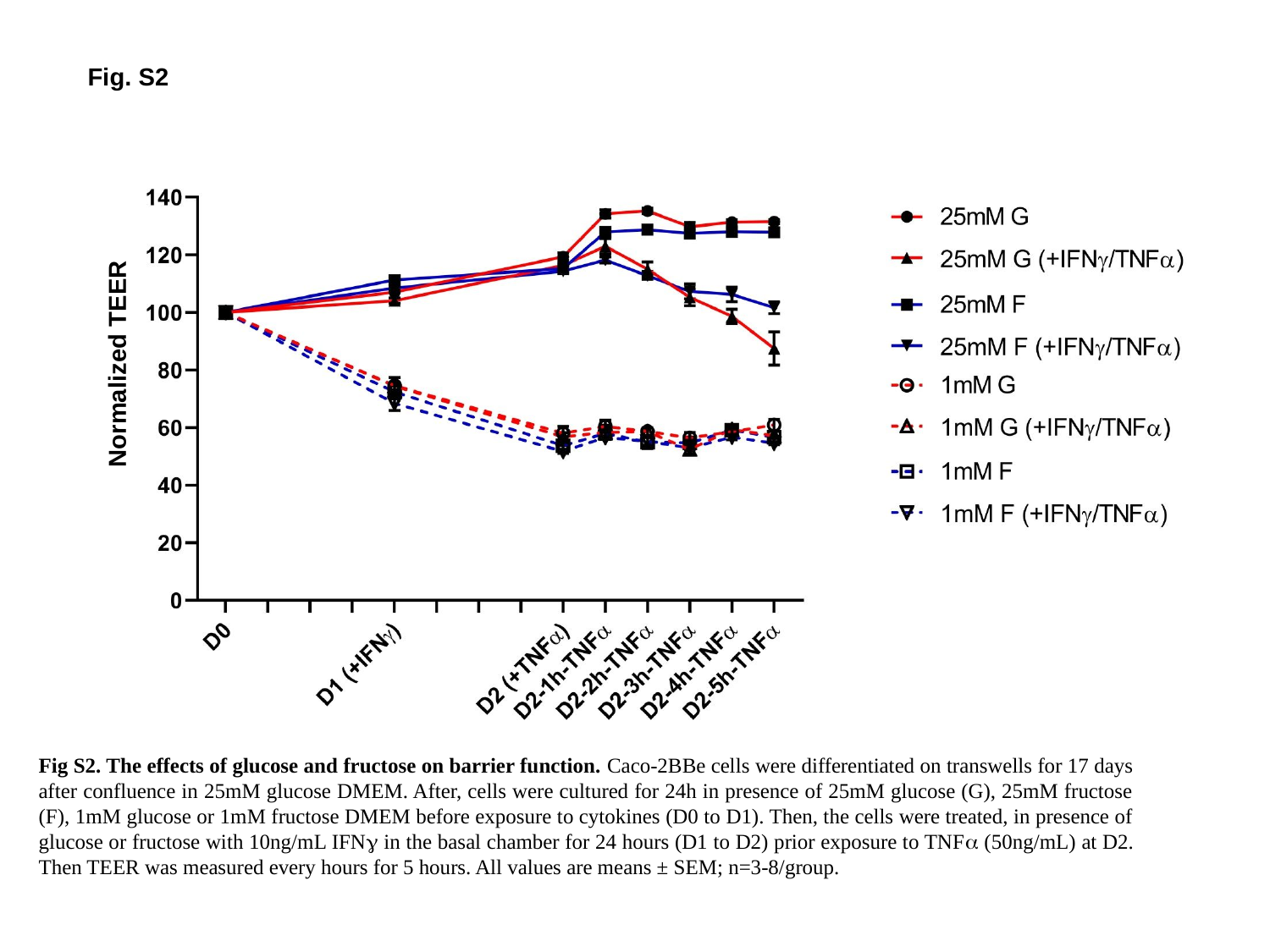

Fig. S2
Normalized TEER
Fig S2. The effects of glucose and fructose on barrier function. Caco-2BBe cells were differentiated on transwells for 17 days after confluence in 25mM glucose DMEM. After, cells were cultured for 24h in presence of 25mM glucose (G), 25mM fructose (F), 1mM glucose or 1mM fructose DMEM before exposure to cytokines (D0 to D1). Then, the cells were treated, in presence of glucose or fructose with 10ng/mL IFNγ in the basal chamber for 24 hours (D1 to D2) prior exposure to TNFα (50ng/mL) at D2. Then TEER was measured every hours for 5 hours. All values are means ± SEM; n=3-8/group.

## Slide 3
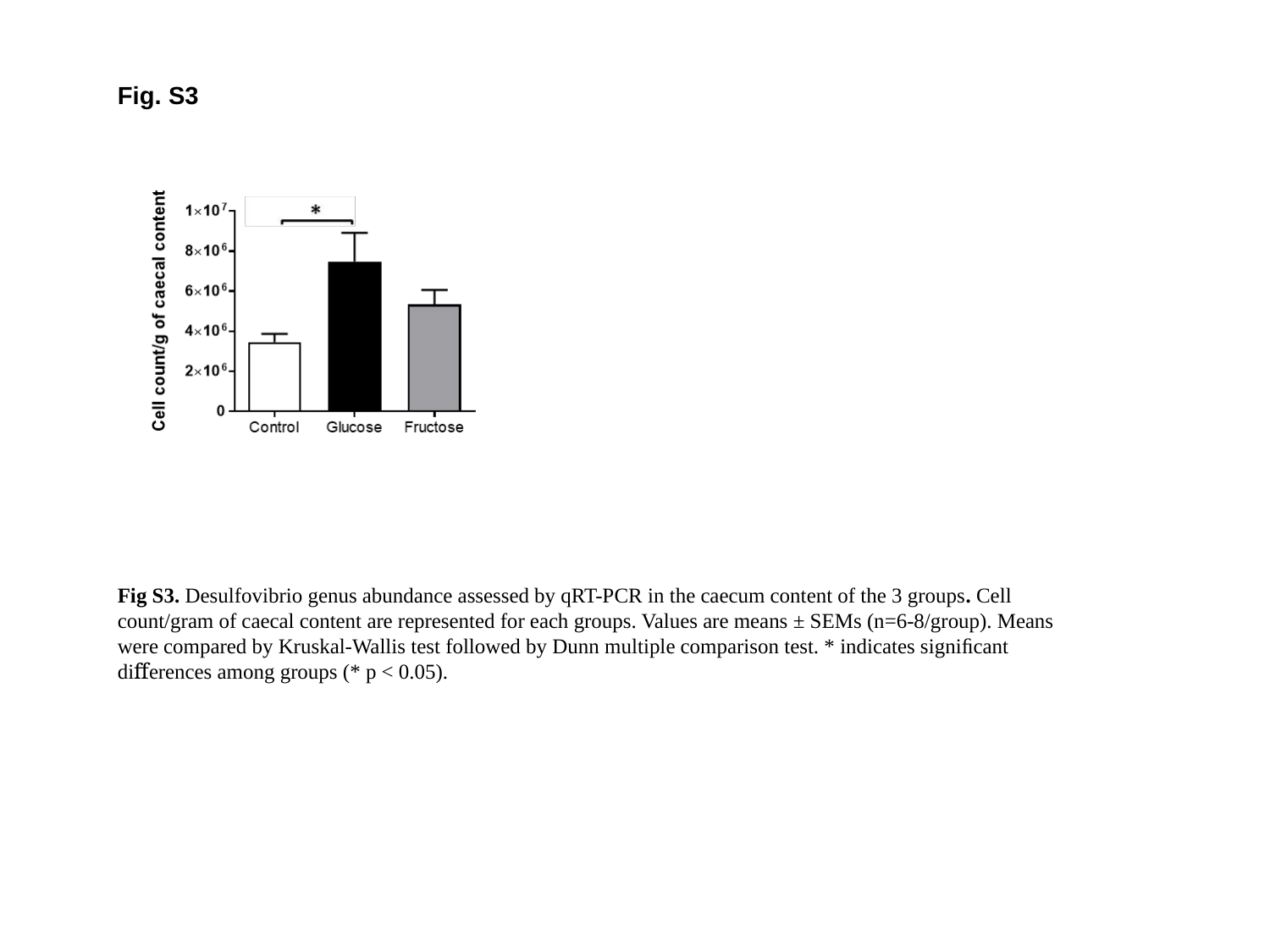

Fig. S3
Fig S3. Desulfovibrio genus abundance assessed by qRT-PCR in the caecum content of the 3 groups. Cell count/gram of caecal content are represented for each groups. Values are means ± SEMs (n=6-8/group). Means were compared by Kruskal-Wallis test followed by Dunn multiple comparison test. * indicates signiﬁcant diﬀerences among groups (* p < 0.05).

## Slide 4
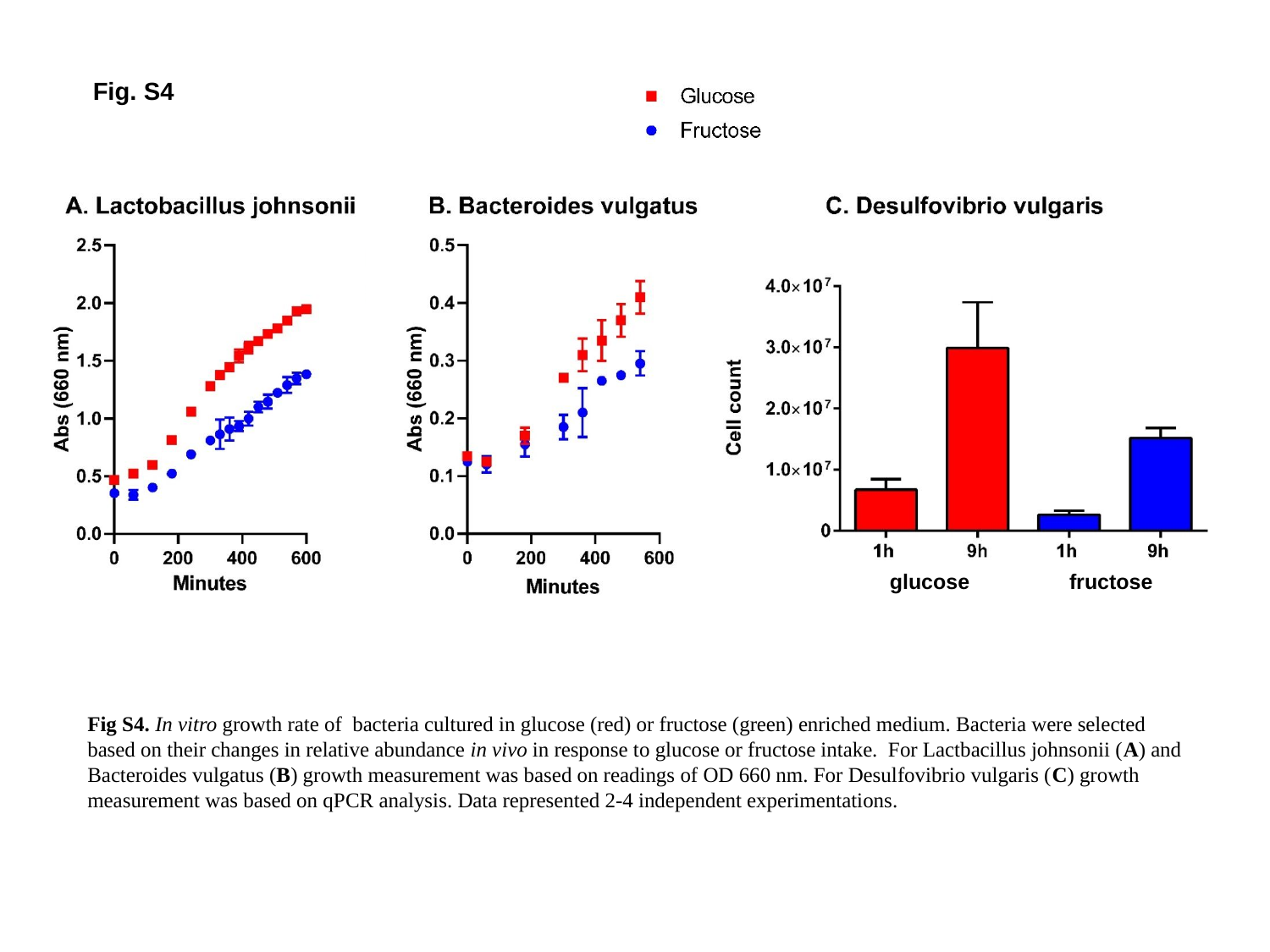

Fig. S4
glucose
fructose
Fig S4. In vitro growth rate of bacteria cultured in glucose (red) or fructose (green) enriched medium. Bacteria were selected based on their changes in relative abundance in vivo in response to glucose or fructose intake. For Lactbacillus johnsonii (A) and Bacteroides vulgatus (B) growth measurement was based on readings of OD 660 nm. For Desulfovibrio vulgaris (C) growth measurement was based on qPCR analysis. Data represented 2-4 independent experimentations.

## Slide 5
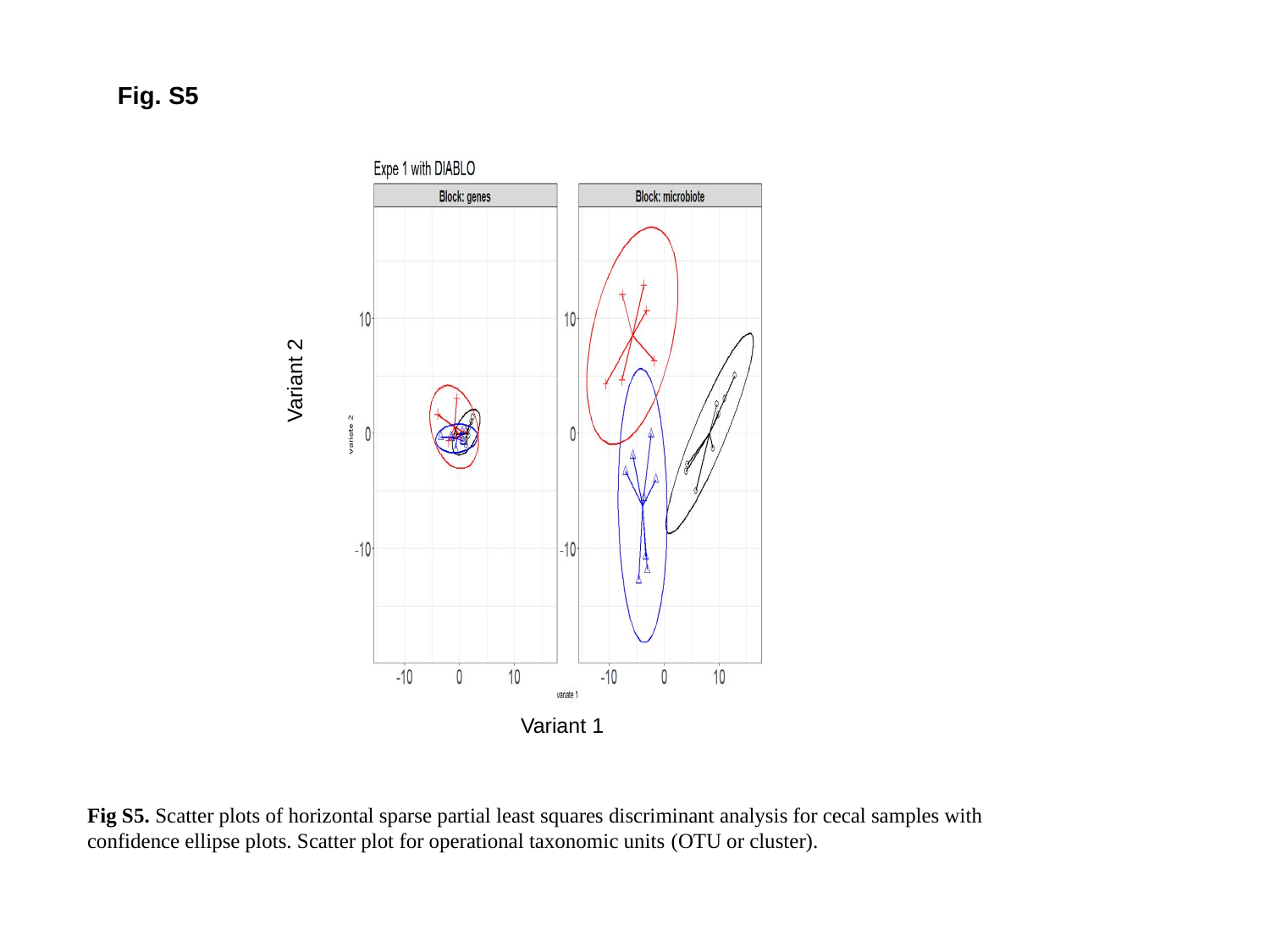

Fig. S5
Variant 2
Variant 1
Fig S5. Scatter plots of horizontal sparse partial least squares discriminant analysis for cecal samples with confidence ellipse plots. Scatter plot for operational taxonomic units (OTU or cluster).
